# Astrocytes as a Therapeutic Target in Alzheimer’s Disease–Comprehensive Review and Recent Developments

**DOI:** 10.3390/ijms232113630

**Published:** 2022-11-07

**Authors:** Mateo Rodríguez-Giraldo, Rodrigo E. González-Reyes, Sofía Ramírez-Guerrero, Carlos E. Bonilla-Trilleras, Santiago Guardo-Maya, Mauricio O. Nava-Mesa

**Affiliations:** Grupo de Investigación en Neurociencias (NeURos), Centro de Neurociencias Neurovitae-UR, Instituto de Medicina Traslacional (IMT), Escuela de Medicina y Ciencias de la Salud, Universidad del Rosario, Bogotá 111711, Colombia

**Keywords:** Alzheimer’s disease, astrocytes, neurodegeneration, glial cells, excitotoxicity, neuroinflammation, oxidative stress, amyloid, therapeutics

## Abstract

Alzheimer’s disease (AD) is a frequent and disabling neurodegenerative disorder, in which astrocytes participate in several pathophysiological processes including neuroinflammation, excitotoxicity, oxidative stress and lipid metabolism (along with a critical role in apolipoprotein E function). Current evidence shows that astrocytes have both neuroprotective and neurotoxic effects depending on the disease stage and microenvironmental factors. Furthermore, astrocytes appear to be affected by the presence of amyloid-beta (Aβ), with alterations in calcium levels, gliotransmission and proinflammatory activity via RAGE-NF-κB pathway. In addition, astrocytes play an important role in the metabolism of tau and clearance of Aβ through the glymphatic system. In this review, we will discuss novel pharmacological and non-pharmacological treatments focused on astrocytes as therapeutic targets for AD. These interventions include effects on anti-inflammatory/antioxidant systems, glutamate activity, lipid metabolism, neurovascular coupling and glymphatic system, calcium dysregulation, and in the release of peptides which affects glial and neuronal function. According to the AD stage, these therapies may be of benefit in either preventing or delaying the progression of the disease.

## 1. Introduction

Alzheimer’s disease (AD) is the most common form of dementia (accounting for 60% to 80% of all dementias), and the most frequent neurodegenerative disease in the aging population [1,2]. AD is a devastating neurological disorder that progressively impairs cognition and memory, severely affecting the quality of life and well-being of patients, family members and caregivers [3]. AD is a multifactorial disease characterized by selective neurodegeneration, abnormal amyloid beta (Aβ) deposits and intracellular fibrillary tangles composed of hyperphosphorylated tau protein [4]. Before amyloid burden, it has been proposed that prefibrillar oligomers affect calcium homeostasis and synaptic transmission [5]. The development of all these pathological features correlates with the clinical appearance of cognitive impairment and dementia [6]. In addition, exacerbated neuroinflammation and oxidative stress have also been observed in brains from individuals with AD, and are current topics of interest in the pathophysiological research of this disease [7]. Various modifiable (diet, lack of exercise, etc.) and non-modifiable (i.e., advanced age) risk factors have been reported for AD [8]. Among the non-modifiable risk factors, several genetic alterations have been associated with either early-onset (familial) or sporadic/late-onset AD forms [9]. For instance, the presence of apolipoprotein E ε4 (APOE-ε4) allele, is the main genetic risk factor for late-onset disease [10], while early-onset disease is more frequently caused by mutations in the amyloid precursor protein (APP), presenilin 1 (PSEN1) and 2 (PSEN2) genes [11].

Astrocytes are the main homeostatic regulators of the central nervous system (CNS) [12]. These cells have crucial functions involving numerous physiological aspects of the brain and spinal cord, including the supply of metabolic substrates to neurons and other glial cells [13], the regulation of ions and neurotransmitters in the extracellular space [14], the release of gliotransmitters [15], and the modulation of synaptic activity [16], among others. Moreover, astrocytes provide intrinsic neuroprotection to neurons [17], and currently, these neuroprotective features are being explored as potential therapeutic interventions in numerous conditions such as stroke and neurodegeneration [18,19]. In addition, astrocytes are functional constituents of the blood–brain barrier (BBB), the neurovascular unit (NVU) [20], and the glymphatic system [21].

The role astrocytes play in AD is a growing topic of interest due to the involvement of these cells in the pathophysiological processes underlying this disease [22,23]. Recent research has highlighted the astrocyte’s involvement in neuroinflammation and oxidative stress in AD [24], the beneficial and detrimental aspects of astrocyte reactivity in AD pathophysiology [25], the astrocytic-amyloid relationship [26], and the importance of astrocyte-neuron interactions in AD pathophysiological processes [27,28]. In AD, various astrocytic functional processes such as calcium signaling, glutamate clearance, extracellular potassium buffering, and energetic metabolism are compromised [29]. In addition, many phenotypic changes have been reported in astrocytes in AD, depicting a large cellular heterogeneity in this disease [30]. In AD experimental mice models, it has been shown that astrocytes migrate in response to chemokines such as monocyte chemoattractant protein-1 (MCP-1), present in Aβ plaques, internalizing and degrading amyloid peptides [31,32]. Moreover, astrocytes, together with microglia, seem to be the drivers of the augmented neuroinflammatory response reported in AD [33]. Furthermore, a recent study showed that glypican-4 (a binding partner of APOE-ε4) secreted by astrocytes, induced tau hyperphosphorylation in a mouse tauopathy model [34]. These findings support an important participation of astrocytes in AD and highlight the need to broaden our comprehension of AD pathogenesis involving glial cells. This approach may allow the identification of specific astrocytic biomarkers for AD [35], and the possibility of developing AD novel therapeutics based on astrocyte’s pathways and functions [36,37]. The possible therapeutic value of astrocytes in AD due to their role in senescence, neuroinflammation, neurotrophic factor release and Aβ clearance, has been widely described [38,39]. In the present article, we will review the effects of both known and novel molecules which act on astrocytes, mentioning new developing therapeutics and pharmacological targets in oxidative stress, neurovascular coupling, excitotoxicity, APOE modulation, among many others, and the way these possible therapeutic approaches could modify either disease instauration or AD progression. In addition, non-pharmacological therapies that impact astrocyte activity will be discussed briefly.

## 2. Astrocytic Role in AD Pathophysiology: Recent Developments

Astrocytes are highly active cells that respond to both physiological and pathological challenges [39]. Reactive astrocytes (RA) have the ability to adopt distinct states, influencing both transient and long lasting changes in deleterious processes [25,40]. Characteristically, RA have an increased expression of glial fibrillary acidic protein (GFAP), which is also overexpressed in AD in response to Aβ [41]. Two contrasting gene expression profiles that can be induced in RA have been proposed: the first one, termed A1, with predominant neurotoxic properties, and the second, A2, with mostly neuroprotective functions [25]. A1 profile has been commonly expressed in neurodegenerative diseases like AD [42]. For this A1 profile to be induced, microglia must secrete the cytokines interleukin-1α (IL-1α), tumor necrosis factor (TNF), and the complement component 1, subcomponent q (C1q) [42]. However, some researchers believe that this binary classification is insufficient, suggesting a broader spectrum for RA based on molecular and functional parameters [40]. RA have been detected in the prodromal phases of AD [35], and in those with elevated Aβ load [43], suggesting that astrocyte reactivity occurs as an early phenomenon in AD pathogenesis. Furthermore, a recent study showed that conditioned media derived from AD transgenic (3xTg-AD) old astrocytes (but not from young ones) had neurotoxic effects in vitro [44]. These effects were found to involve a proapoptotic mechanism, dependent on glycogen synthase kinase-3β (GSK3β) activation (a kinase involved in tau hyperphosphorylation), and a proinflammatory response. Similarly, decreased calcium signaling and reduced IP3 receptor type 2 (IP3R2) expression in astrocytes was reported in human brains from individuals with AD, and associated with early alterations of functional connectivity and network activity in the APPNL-F mice model for AD [45]. In the same study, neuronal hyperactivity, seizure susceptibility, behavioral disruptions and abnormal functional connectivity were corrected when astrocytes returned to normal calcium signaling. In addition, astrocytes have been shown to be involved in many other aspects of AD such as amyloid and tau protein metabolism [46], neuroinflammation and oxidative stress [23], and alteration in gliotransmission and neurotransmission [47,48], including excitotoxicity [49], among others. These observations allude to an important role of astrocytes in AD pathogenesis and development and assert the need to investigate therapeutic agents for AD involving these glial cells.

### 2.1. Astrocytes in Tau Protein and Amyloid Metabolism

The “amyloid hypothesis” states that the accumulation of Aβ in the brain is responsible for the development of AD and the associated behavioral and cognitive symptoms [50]. This hypothesis has been at the forefront of AD research for the past decades. However, its potential as a therapeutic target in AD pathogenesis remains controversial [51,52]. Nonetheless, it has been reported that astrocytes and amyloid peptides share a connection in AD. For instance, astrocytes have been shown to degrade Aβ plaques through enzymatic cleaving [53], and also to internalize Aβ_1–42_ both in vitro and in vivo in mice [31], and in vitro in human cells [54,55]. Furthermore, astrocytic activation (coinciding with beta-secretase-1 (BACE1) activity) has been detected before amyloid deposition in transgenic mice models with overexpressed mutant APP [56]. After stimulation with interferon gamma (IFN-γ) and TNF, astrocytes increased the levels of BACE1 and APP, with a consequent increase in Aβ production [57]. Although this implies that RA could be considered as a major source of Aβ in the neuroinflammatory context of AD, astrocytes may also have neuroprotective effects at the early stages of amyloid production.

APOE is involved in the homeostatic control of plasma and tissue lipid content, and possesses three major isoforms, APOE-ε2, APOE-ε3 and APOE-ε4, of which APOE-ε3 is the most commonly expressed [58]. Under normal conditions, astrocytes, together with microglia, are the main producers of APOE in the CNS [59]. However, the expression and secretion of APOE can be affected in an isoform- and cell-manner by the presence of inflammation [60]. In fact, human astrocytes carrying the APOE-ε4 allele manifest a pro-inflammatory phenotype, with increased activity of the nuclear factor kappa-light-chain-enhancer of activated B cells (NF-κB) [61]. The APOE-ε4 isoform has been considered as an important genetic risk factor for developing sporadic late-onset AD (SAD) [62], and is currently being explored as a biomarker for AD risk [63]. Research in AD transgenic mice, suggest that the pathogenic role of APOE-ε4 involves both a noxious gain of function after interacting with Aβ, and a loss of protective functions that alter astrocyte activation, precipitating synaptic loss [64]. Moreover, human carriers of the APOE-ε4 allele present more Aβ plaques than those with APOE-ε3 or other isoforms [65]. Interestingly, the specific removal of APOE-ε4 in astrocytes decreases phosphorylated tau and tau-associated neurodegeneration [66]. These findings provide a link between the APOE-ε4 allele and AD pathogenesis, suggesting that astrocyte-derived APOE-ε4 can be a regulator of tauopathies [67].

AD is considered a secondary tauopathy as it has other pathogenic drivers beside tau deposition (mainly the aforementioned Aβ) [68]. Under pathological conditions, tau is hyperphosphorylated, causing cytosolic aggregation of paired helical filaments and consequently neurofibrillary tangles, which are considered one of the features of AD [69]. Although AD shows predominantly neuronal tau pathology, thorn-shaped astrocytes with perinuclear tau deposits have also been reported [70,71], particularly in aging-related tau astrogliopathy models [72]. Furthermore, the presence of astrocytes with abnormal tau aggregates are correlated with neurodegeneration, suggesting that astrocytes are capable of internalizing this protein [73]. A recent transcriptomic study in post mortem human brains with AD showed that differences in glial gene expression were correlated with tissue amyloid or phosphorylated tau expression, with astrocytes showing enrichment for proteostatic, inflammatory and metal ion homeostasis pathways [74]. In fact, single-nuclei RNA-sequencing transcriptomics in post-mortem human brains with AD identified risk loci associated with astrocytic-mediated tauopathy, such as the genes for clustering (CLU), myocyte enhancer factor 2C (MEF2C) and IQ domain-containing protein K (IQCK) (a calcium binding protein whose function remains unclear) [74]. CLU, also called apolipoprotein J, is an extracellular chaperone that strongly binds to tau promoting internalization through endocytosis, possibly accelerating the spread of tau pathology [75]. Interestingly, the CLU gene is one of the most important genetic risk factors for late onset AD [75,76]. Moreover, increased expression of CLU was associated with phosphorylated tau pathology in astrocytes [74]. MEF2C is an AD risk-associated transcription factor consisting of four proteins that play a central role in pathways affecting neuronal development [77]. MEF2C mutations have been described as a genetic cause of developmental disorders [78], and genome-wide association studies (GWAS) have shown that it is involved in AD risk and progression [79,80,81]. In the case of IQCK, it was found to be highly expressed in astrocytes, neurons and oligodendrocytes (but not in microglia) in AD human brains [82]. However, other genes may also be involved in the relationship between Aβ and tau. For example, the WW domain-containing oxidoreductase (WWOX) gene, a high-density-lipoprotein cholesterol and triglyceride–associated gene which binds tau and has been involved in the regulation of tau hyperphosphorylation, neurofibrillary tangle formation and Aβ aggregation, was found to be mainly expressed in astrocytes and neurons from the brains of individuals with AD [83]. Moreover, the astrocytic cyclin-dependent kinase 2-associated protein 1 (CDK2AP1) has shown a significant association with cognitive function examinations (Mini-Mental state examination test), neurofibrillary tangles and amyloid plaques in human AD studies [84].

Tau uptake in astrocytes is mediated by the integrin complex alphaV/beta1 [85], followed by lysosomal degradation or glymphatic clearance [67,86]. Astrocytic tau accumulation in the dentate gyrus of individuals with AD has been shown to disrupt mitochondrial function, thereby, impairing neurogenesis, reducing the number of interneurons, decreasing the density of inhibitory synapses and reducing gamma oscillatory activity [87]. Furthermore, in the same paper, the authors observed that injection of neuregulin 1 peptide (NRG1p) was able to rescue spatial memory impairments due to astrocytic tau accumulation in C57BL/6 mice. Other researchers have identified astrocyte internalization of extracellular tau oligomers with a corresponding alteration in intracellular calcium signaling, hindered calcium gliotransmission and a compromise in ATP release [88]. These effects resulted in changes on pre- and postsynaptic protein expression, and modifications in synaptic activity in adjacent neurons. Moreover, tau accumulation in astrocytes can induce the release of tau oligomers, precipitating propagation and spread of that protein through the exosomal-dependent pathway [73]. In addition, astrocytes contain tunnelling nanotubules, contributing to prion-like transfer and propagation of alpha-synuclein [89]. Thus, this same route of cytoplasm-to-cytoplasm transfer could also be used to deliver tau from astrocytes to other cells in a similar manner to the intracellular propagation of tau in neurons [73].

In summary, astrocytes participate in the internalization and degradation of Aβ; however, their abnormal activation under a pro-inflammatory context, leads to an increase in Aβ production and release. This effect is also associated with the spread and accumulation of tau within the astrocyte, affecting its proper functioning. Several genes widely expressed in astrocytes (i.e., APOE-ε4, WWOX, CLU and CDK2AP1, among others) are related to AD risk and progression, and also have been implicated in the joint metabolism of Aβ and tau. Therefore, activation of specific astrocyte pathways could be a key factor to promote Aβ and tau clearance at early stages of AD progression. Amyloid clearance therapies based on astrocytic modulation will be reviewed in Section 3.

### 2.2. Astrocytes, Neuroinflammation and Oxidative Stress

Astrocytes are involved in neuroinflammatory processes through activation of intracellular pathways such as the NF-κB pathway, the production of nitric oxide (NO) and reactive oxygen species (ROS), and the release of various proinflammatory cytokines including IL-1β, IL-6 and TNF [24]. In addition, other factors can induce the production of proinflammatory cytokines such as the glia maturation factor (GMF), which has been found to be upregulated in AD [90]. However, astrocytes can also express and release anti-inflammatory and neuroprotective factors [91]. Therefore, astrocytes may play a central role in the neuroinflammatory processes observed in AD. Furthermore, impaired astrocytic regulation increases cytotoxicity and oxidative stress, inducing production and aggregation of Aβ plaques in AD [92]. In addition, there is evidence showing that Aβ_1–42_ oligomers can induce oxidative stress via binding to the receptor for advanced glycation end products (RAGE) on astrocytes, and to induce ROS production through the activation of the NADPH oxidase (NOX) complex [93,94]. Thus, astrocytes are involved in two important pathological aspects of AD such as neuroinflammation and oxidative stress.

In AD, astrocytes and microglia are intimately related in the pathophysiological processes of the disease. Experimental models of neuroinflammation show that glial activation occurs at different stages, with an earlier activation of microglia followed by an astrocytic activation response [95]. Recently, in the 5xFAD mouse model for AD, it was shown that communication between microglia and astrocytes is mediated by IL-3, produced by the latter, which instructs microglia to cluster around the Aβ plaques in order to clear them, thus reducing Aβ load, and improving memory [96]. Moreover, some studies have shown that IL-3 levels have been positively correlated with both AD risk and disease severity [97,98,99]. Calcium dysregulation is another important aspect observed in AD, which involves a crosstalk between astrocytes and microglia. A bidirectional relationship between hyperactivity of calcium channels and calcium-calcineurin pathway with the subsequent release of proinflammatory cytokines from glial cells have been described in animal models of aging and in neurodegenerative conditions [100,101]. These effects not only impact astrocyte activity, but also may affect synaptic plasticity and excitability in neurons.

Oxidative stress develops due to an imbalance between the production of free radicals over antioxidants in the mitochondria [102]. In AD, oxidative stress appears secondary to mitochondrial dysfunction, which may lead to synaptic Aβ-induced damage [103,104]. This has been linked to increased levels of ROS and reactive nitrogen species (RNS) [105]. Under physiological conditions, astrocytes are fundamental for neuronal antioxidant production, since they synthesize and deliver amino acids such as glycine and cysteine for glutathione (GSH) production in neurons [106,107].

GSH is one of the main endogenous antioxidant agents in the CNS. It is synthesized mainly in astrocytes and is then released to the neuron through the ABCC1 transporter [108]. For this reason, in oxidative stress conditions such as AD, the activity and expression of this transporter is increased to compensate for the antioxidative function. Aβ deposits can alter the antioxidant function of astrocytes mediated by GSH, although this may depend on the amyloid form (i.e., soluble or aggregated), and the duration of exposure [109]. For instance, in vivo and in vitro studies have shown that monomeric forms of Aβ increase ABCC1 expression in acute and late stages of AD [108,110]. However, one of those studies showed that prolonged incubation with fibrillary forms of Aβ reduces expression of the ABCC1 transporter in 5xFAD mice cortices [107]. Therefore, in amyloidosis models, ABCC1 transport activity is probably increased by an induction of its expression in astrocytes that promotes GSH release as a protective mechanism against oxidative stress, although this mechanism is not sustained in the long-term. It has been observed that the Aβ isoforms Aβ_1–42_ and Aβ_25–35_ increase hydrogen peroxide (H_2_O_2_) production and GSH release in astrocytes [111]. In fact, Aβ levels have shown to be directly correlated with ROS production, with large amounts of ROS inducing a neurotoxic profile in astrocytes, through the expression of inducible nitric oxide synthase (iNOS), causing nitrosative stress and toxic nitration in neurons [25]. The process of astrocytic iNOS stimulation due to Aβ has been shown to be dependent of IL-1β and TNF, through a NF-κB inducing kinase (NIK)-dependent signaling mechanism [112]. These findings suggest that the induction of neurotoxic versus neuroprotective RA profiles are correlated with the level of ROS production.

There is a continuous cycle between neuroinflammation and oxidative stress, in which one of these pathological processes induces the other and feeds-back positively. The way both oxidative stress and neuroinflammation interact in the astrocyte is exemplified by the RAGE-NF-κB pathway, an intracellular pathway which can be activated by Aβ. The antioxidant effects in which astrocytes participate through the production and release of GSH could have a neuroprotective role in early stages of AD. However, prolonged exposure to amyloid may cause an impairment in these physiological mechanisms, increasing the production of free radicals. Finally, this abnormal chronic activation of astrocytes promotes brain inflammation through cytokine interactions with microglial cells.

### 2.3. Astrocyte Role in Gliotransmission and Excitotoxicity

Astrocyte participation in memory and other cognitive processes is essential, since both synaptic transmission and plasticity depend on the astrocytic control of gliotransmitters and neurotransmitters involved in excitatory neurotransmission (mainly glutamate), necessary for the regulation of both long-term potentiation (LTP) and long-term depression (LTD) [113]. Astrocytes are also involved in the structural remodeling and functional plasticity of synapses [16]. Thus, abnormal astrocytic signaling is implicated in cognitive impairment in AD, even at prodromal or early AD stages [15]. These findings have made these cells an interesting new therapeutic target in the recovery of cognitive functions before neurodegeneration is established [113].

Astrocytes are engaged in the regulation of synaptic function through the release of gliotransmitters such as glutamate, glycine, gamma-aminobutyric acid (GABA), D-serine and ATP [114]. Cumulative and recent evidence highlight the role of astrocytic GABAergic systems in the pathophysiology of AD, as well as a possible target for pharmacological interventions (for review see [115]). Several human studies and animal models indicate an increase in GABAergic function and GABA contents in RA close to amyloid plaques, associated with memory impairment [116,117]. Correspondingly, it has been proposed that selective antagonists of astrocytic GABA in determined brain regions could reduce cognitive deterioration in AD, though this blocking could be technically difficult. On the other hand, considering the anti-inflammatory effects of GABA receptors (GABA A and B) activation in cultured human astrocytes [118], and their role in the control of neuronal hyperexcitability, it has been proposed that this effect may be a compensatory mechanism to reduce neurodegeneration at early stages of AD [116]. For this reason, the therapeutic modulation of the GABAergic system will depend on the AD stage, the affected brain region, and the specific circuit dysfunction. The glutamate/GABA-glutamine cycle (a metabolic pathway in which the astrocyte plays a main role) is also affected in AD, and could be an interesting target of modulation [119]. Furthermore, it has been shown that the GABAB-G protein-coupled inwardly rectifying potassium (GIRK) channel activity is reduced by Aβ in the hippocampus of mice and rats [115,120], and that GIRK activation may prevent both synaptic plasticity and memory impairments in amyloidosis in vivo and ex vivo models [121,122]. Therefore, GIRK represents a very interesting therapeutic target, taking into account its fundamental role in hippocampal function and, therefore, in memory [123]. Likewise, several types of inwardly rectifying potassium channels are expressed in astrocytes (i.e., Kir3.1 protein and Kir4.1-encoded by KCNJ10 gene) [124], but their role in the pathophysiology of AD is still unknown.

Excitotoxicity is associated with dysregulation in astrocyte–neuron signaling pathways [29]. In AD, excitotoxicity is considered a multicausal event arising from the combined effects of dysfunctional glutamate re-uptake and enhanced glutamate release, leading to overactivation of NMDA receptors, synaptic loss, and consequently, neuronal death [125]. Under physiological conditions, astrocytes remove about 90% of all released glutamate in the CNS through the excitatory amino acid transporters 1 (EAAT1, also referred to as GLAST-1) and 2 (EAAT2, also referred to as GLT-1) [126]. During AD progression, there is a reduction in astroglial glutamate uptake that predisposes to excitotoxicity [127,128]. In the APPSw, Ind. mice AD model, restored EAAT2 function through a pharmacological intervention (LDN/OSU-0212320), led to an improved cognitive function, a decrease in amyloid plaques and a recovery of synaptic integrity [129]. Therefore, astrocytic regulation of glutamate through EAAT’s can be considered as a therapeutic target for the reduction or prevention of excitotoxicity in AD.

Astrocytes are deeply involved in CNS cellular signaling, including a very tight control of synaptic glio and neurotransmission. In AD, both the excitatory and inhibitory neurotransmitter systems are compromised. This leads to a poor regulation of synaptic activity, in particular at the postsynaptic neuron, where these alterations can lead to either excessive excitation or poor inhibition. For instance, the expression of EAATs is reduced in AD, diminishing the capacity of the astrocyte to reuptake glutamate from the synaptic cleft, thus increasing the possibility of excitotoxicity.

## 3. Astrocytes as a Therapeutic Target: Treatment and Neuroprotection Strategies Based on Astrocyte Modulation

The neurocentric view of AD pathogenesis has proven to be an insufficient explanation for the disease’s origin, and has restricted the development of new therapeutics [25]. Despite billions of dollars spent on AD research, there is a limited number of available therapies. One of these, is the United States Food and Drug Administration (FDA) approved aducanumab that targets Aβ, but it’s very expensive, and the two clinical trials that granted FDA approval failed to show a conclusive benefit [130]. However, in a recent phase III clinical trial (NCT03887455), a promising result was reported with lecanemab, an anti-Aβ protofibril antibody that may reduce cognitive decline in patients with early AD [131]. One explanation of the success of lecanemab may be due to an earlier intervention compared with other studies, and because it was well tolerated by the participants. Since neuronal death is a final cellular event of AD, there is a need to find therapeutic targets based on the sequence of events of AD progression before neurodegeneration ensues. The controversies surrounding the traditional AD models, and the recent interest in astrocytic physiology and its role in neurological diseases, show that astrocyte pathways and functions can be promising therapeutic targets in many CNS conditions.

Interest in astrocytes as a therapeutic target in AD arises from the multiple ways in which they interact with known pathological processes underlying the disease, as described previously. Considering that astrocytes are involved in multiple homeostatic pathways, it is difficult to target one dysregulated pathway during AD progression without affecting physiological or compensatory responses. In the present section we will review the therapeutic approaches targeting astrocytes in AD regarding their main action mechanism. 

### 3.1. Neuroinflammatory Control

Neuroinflammation has been linked to several neurodegenerative disorders, especially AD. Recently, new evidence has shown that targeting inflammatory mechanisms may alter disease progression in AD models through different pathways, such as the promotion of Aβ-clearance, the release of trophic factors, antioxidants and metabolites, or through a decrease in ROS generation [25]. As we mentioned before, the crosstalk between astrocytes and microglia represents another important pathway to develop or maintain a neuroinflammatory response. Recently, IL-3 produced by astrocytes provided an important clue of how this cellular interaction between astrocytes and microglia may occur in AD [96]. As IL-3 signaling has been shown to direct microglia to reduce Aβ burden and improve memory in animal models, it can be proposed as a novel therapeutic intervention for AD. However, more studies are required since this approach depends on the delivery mode, as peripheral IL-3 injection showed no effect on AD pathology.

In CNS, the complement component 3 (C3) is an astrocyte-released factor that may induce neuronal damage via C3aR1 receptor. The expression of C3 and C3aR1 was found to be positively correlated with cognitive decline and Braak staging in brains from individuals with AD, suggesting that complement activation is correlated with AD pathology and disease severity [132]. In the same paper, the authors reported that a deletion of C3aR1 in PS19 mice was able to reverse neurodegenerative features, rescue tau pathology and reduce neuroinflammation. Interestingly, astrocyte-released C3 also mediates the crosstalk between astrocytes and microglia, indicating a possible continuous cycle among neurons, astrocytes, and microglia in tauopathy [67,133]. Considering that C3 overexpression activates the Janus kinase 2 (JAK2)/signal transducer and activator of transcription 3 (STAT3) pathway [134], and that intracellular signaling is crucial in astrocyte reactivity and proliferation [135], it could be an interesting modulatory target for AD. Indeed, it has been shown that astrocytic STAT3 overexpression induces neuroinflammation and neuronal death after Aβ-oligomers microinjection in rats [136]. In addition, a study found that daphnetin (a coumarin derived from the *Daphne giraldii* plant) could improve cognitive function in the APP/PS1 mouse model of AD, possibly by inhibiting STAT phosphorylation [137]. Considering that phosphorylated STAT3 (p-STAT3) can enter the nucleus and bind to the GFAP promoter to induce GFAP expression, this effect may limit astrocytes activation [138]. In addition, STAT3 deletion in the APP/PS1 mice model was related to smaller Aβ plaques, increased Aβ clearance and reduced pro-inflammatory cytokine release [139]. In an AD model induced by intracerebroventricular streptozotocin delivery in Wistar rats, it has been shown that kaempferol reduced cognitive impairment, specifically through antioxidative mechanisms such as an increase in the levels of superoxide dismutase (SOD) and GSH [140]. Although it was not tested in a specific AD model, kaempferol reduced the activation of complement C3 and the neurotoxic astrocyte profile induced by 3-nitropropionic acid in rats [141]. The mechanism proposed by the same authors indicates an anti-inflammatory effect through reduced expression of NF-κB, which prevented the production of amyloid in the striatum and hippocampus. Neuroprotective effects of kaempferol have been also tested in focal stroke, where this molecule may inhibit the activation of STAT3 [142]. Furthermore, the STAT pathway proteins can be regulated by members of the STAT-induced STAT inhibitor (SSI) family, such as the suppressor of cytokine signaling 3 (SOCS3) [143]. Astrocytic expression of SOCS3 and modulation of JAK2/STAT3 by this pathway, resulted in reduced amyloid deposition, improved spatial learning and attenuation of neuroinflammation in APP/PS1dE9 transgenic mice [144]. A study in mice found that low-dose aspirin can up-regulate SOCS3 in astrocytes and microglia by binding to the peroxisome proliferator-activated receptor alpha (PPARα) both in vitro and in vivo, with SOCS3 expression increased in GFAP-positive astrocytes, suggesting that aspirin can be a potent anti-inflammatory therapeutic target in AD [145].

A study conducted in rat neuronal and astrocytic cultures showed that the accumulation of RA in response to Aβ-induced neuroinflammation increases caspase-3 activation, tau production and neuronal death [146]. In the same study, through the use of minocycline, a tetracycline antibiotic with anti-inflammatory properties, authors reported a decrease in astrocytic and caspase-3 activation, with a consequent reduction in neuronal loss. Furthermore, the administration of minocycline in a tau mouse model, reduced the number of activated astrocytes, decreased the formation of abnormal tau species, and attenuated the production of proinflammatory cytokines [147].

The upregulation of cyclooxygenase-2 (COX-2) and prostaglandins (PGs) contribute to the pathogenesis of AD, particularly through PGE2 and PGI2, by inducing Aβ production and triggering hyperphosphorylation of tau protein [148]. DP1, a prostaglandin receptor, is overexpressed in astrocytes and microglia in amyloid plaques in both AD patients and Tg2576 mice [149]. This prostaglandin receptor has shown neuroprotective effects against glutamate-induced neurotoxicity [150]. Physiologically, DP1 activation increases in response to toxicity; therefore, as Aβ deposition occurs and neuroinflammation settles, DP1 activity rises in compensation [151]. Contrastingly, activation of DP2 receptors exacerbates neuroinflammation via astrogliosis [152]. Although there are no particular studies regarding its neuroprotective action in astrocytes, we propose two potential therapeutic targets to control PGD2-induced neuroinflammation in AD. Firstly, the DP1 agonist, BW245C, which has shown neuroprotective effects against ischemic brain damage [153], and secondly, the DP2 antagonist BWA868C [154], which could possibly slow down neuroinflammation in AD.

1-trifluoromethyl phenyl-3-(1-propionylpiperidin-4-yl) urea (TPPU), a small molecule soluble epoxide hydrolase blocker (sEH), has shown promising results as an AD therapeutic. TPPU can block astrocytic sEH up-regulation, and astrocyte-microglia interaction, with a reduction in LPS-induced inflammation, both in vitro and in vivo [155]. Furthermore, TPPU has shown to reduce oxidative stress damage and induce brain-derived neurotrophic factor (BDNF) expression in the rat cell line PC-12 [156]. Reported evidence showed that TPPU can also reduce inflammatory gene expression, presenting down-regulation of 73 inflammatory genes, with an associated reversion of dysregulation in immune pathways [155]. TPPU can also reduce gliosis, and the number and size of Aβ plaques, with cognitive improvement being reported in the 5xFAD mouse AD model [157]. This suggests sEH as a promising pathway of intervention for future AD therapeutics.

As mentioned in Section 2, several intracellular signaling pathways related with inflammatory responses and oxidative stress are involved in the pathophysiology of AD. For instance, Aβ can activate the NF-κB pathway, responsible for the transcription of pro-inflammatory cytokines and chemokines in astrocytes [158]. In a recent study, it has been shown that isothiocyanates, such as sulforaphene (SF), possess neuroprotective and anti-inflammatory effects through inhibition of both NF-κB pathway and PI3K/Akt/GSK-3β in an AD-like pathology model, induced by streptozotocin [159]. In the same study, it was shown that SF inhibited the phosphorylation of tau protein and improved cognitive deficits. Another astrocytic related component is the nuclear factor erythroid-derived 2 (Nrf2), a transcription factor which contributes to astrocyte homeostasis through expression of antioxidant proteins in response to oxidative and toxic insults [160]. Deletion of Nrf2 is associated with enhanced inflammation while its upregulation decreases pro-inflammatory responses and NF-ĸB transcription [161]. SF prevents oxidative stress induced by glucose and oxygen deprivation in rat cortical astrocytes through stimulation of the Nrf2 pathway [162]. In a cellular model of AD, using mouse N2a/APPswe cells, SF exerted anti-inflammatory and antioxidative effects [163]. SF also decreased amyloid expression and tau phosphorylation, and reduced markers of synaptic damage [164]. In addition, it has been reported that the beneficial effects of SF on Nrf2 activity preferentially occur in astrocytes [165]. The activation of Nrf2 in these glial cells generates neuroprotection against oxidative stress in a mouse model of amyotrophic lateral sclerosis [166]. Currently, a clinical study evaluates the efficacy, safety and related mechanism of SF in the treatment of patients in prodromal and early AD (NCT04213391). A recent study in a transgenic mouse model of AD (3 × Tg-AD), used cornuside (a phytotherapeutic derived from *Cornus officinalis*), reporting anti-inflammatory and neuroprotective effects due to the modulation of astrocyte activity through AKT/Nrf2/NF-κB signaling [167]. These neuroprotective effects may also regulate the phenotypic changes in the astrocytes and help prevent cognitive impairments.

In order to maintain homeostasis in the CNS, astrocytes have developed a plethora of neuroprotective strategies, including the release of growth factors such as BDNF, GDNF, nerve growth factor (NGF), platelet derived growth factor (PDGF) and insulin-like growth factor 1 (IGF1), among others, which are fundamental for brain metabolism, oxidative stress protection and cell viability [168]. Alterations in the function or expression of these molecules have been reported in AD. For example, NGF levels have been found to be reduced in the hippocampus of individuals with AD [169]. In addition, IGF1 signaling has also been reported to be impaired, as reduced active/inactive IGF1 ratio and increased IGF1 receptor expression in post-mortem hippocampal tissue from AD patients was observed [170]. Therefore, growth factors can also be considered as plausible therapeutic targets for this disease. Milk fat globule epidermal growth factor 8 (MFG-E8) is an anti-inflammatory glycoprotein related to the engulfment of apoptotic bodies, primarily expressed by astrocytes in the brain [171,172]. MFG-E8 has shown to attenuate the reactive response of astrocytes through the downregulation of NF-κB and the upregulation of PI3K-Akt in Aβ_1–42_ stimulated mouse astrocyte-microglia co-cultures [173]. In addition, MFG-E8 released from astrocytes has been reported to act on microglia stimulating the endocytosis of Aβ in rat cell culture, suggesting MFG-E8 as a potential therapeutic target in AD [174].

Neuroinflammation is one of the pathological components observed in AD. The chronic persistence of this condition leads to a compromise in the function of different brain cells, mainly astrocytes and microglia. Due to the critical role of astrocytes in CNS homeostatic regulation, an altered stance of these cells represents numerous disturbances in physiological processes, thus prompting the development of new therapeutic interventions. These can be designed to target different aspects of the dysregulated inflammatory pathways observed in AD. For instance, astrocytic IL-3 or TPPU interventions may improve astrocyte-microglia interactions, while intracellular regulation of NF-κB may limit the astrocytic pro-inflammatory response to Aβ. In addition, controlling other deleterious factors such as oxidative stress and excitotoxicity may also be of benefit for the overall inflammatory state. Despite the promising results observed with some of these approaches, most of the works have been conducted on preclinical animal models, which may not necessarily mirror human pathology. Therefore, more studies, including interventions in both human cells and individuals, are needed in order to develop effective therapeutic targets which can be translated into clinical practice in the near future.

### 3.2. Targeting Oxidative Stress

Oxidative stress appears to be increased in AD as a result of both soluble and aggregates of Aβ, neurofibrillary tangles and mitochondrial abnormalities [175]. Treatments focused on the interference of oxidative stress in AD have been proposed as a novel therapeutic strategy, including AGE-inhibitors, increasing levels of antioxidants, stabilizing mitochondrial activity and prevention of ROS formation [103,176]. Likewise, a recent large-scale study (n = 7283 participants) evidenced an association between serum antioxidants levels, such as the carotenoids lutein+zeaxantin and B-cryptoxanthin, either as potential risk or protective factor in all-cause dementia, including AD [177].

Antioxidant systems are classified in two groups: enzymatic (i.e., catalase and glutathione peroxidase) and non-enzymatic that includes molecules obtained from diet such as vitamin C, carotenoids, flavonoids and polyphenols [178]. Neuroprotective effects of those molecules can be explained due to their antioxidant properties, including the inhibition of lipid peroxidation, modulation of enzymatic activity, control of antioxidant gene expression by modulation of redox-sensitive transcription factors and the induction of genes encoding pro-survival, detoxifying, and antioxidant proteins [179]. Many of these non-enzymatic antioxidant systems (such as vitamin E, flavonoids and carotenoids) have demonstrated neuroprotective effects in different AD preclinical models [180,181,182]. For example, experimental evidence showed that vitamin E improves astrocyte survival after glutamate-mediated toxicity in vitro [183]. However, clinical trials have reported limited benefits of alpha tocopherol (a form of vitamin E) in AD [184,185,186]. In this regard, it has been proposed that therapeutic failure of some antioxidants in AD can be attributed to improper dosage, dissimilar time of interventions or the type of antioxidant used, as well as lack of information in how antioxidant systems work [187,188].

Different studies have explored exogenous molecules with antioxidant potential in cultured astrocytes. Phloroglucinol, a polyphenol present in some algae species, inhibits ROS generation and decreases GFAP expression induced by oligomeric forms of Aβ_1–42_. These effects involve increasing levels of superoxide dismutase (SOD), catalase and GSH peroxidase [189]. Nobiletin, a polymethoxylated flavone derived from citrus peels, and resveratrol, a polyphenol present in grapes, promote HO-1 upregulation, an antioxidant which is associated with decline in ROS, reduction in astrocytic GFAP, and improved mitochondrial function [190,191,192]. Another study found that curcumin, a phenolic compound present in the *Curcuma longa* plant, might counteract some effects of oxidative stress such as mitochondrial dysfunction and astrogliosis [193]. Interestingly, the fruit and seeds of *Bactris guineensis*, a rife type of palm from the Caribbean region, appears to have antioxidant molecules (i.e., cyanidin-3-rutinoside and cyanidin-3-glucoside) which protect astrocytes and neurons against oxidative stress [194]. Cholesterol sulphate (a sterol sulphate) activates anti apoptotic pathways such as AKT/Bcl-2, reduces ROS levels and modulates astrocytic metabolism [195]. Calycosin, a molecule present in *Astragalus membranaceus* (a plant used in Chinese medicine) demonstrated, in an in vitro model of oxidative stress induced by H_2_O_2_, the ability to suppress ROS and inflammatory factors production, and to increase the expression of SOD and antioxidant molecules via Nrf2 signaling pathway [196]. Some of the antioxidative molecules described previously have not been tested in specific models of amyloidosis or tauopathy; however, they might have a therapeutic potential in AD. Considering that polyphenols and flavonoids have low BBB permeability, the use of hybrid compounds has been considered to improve the brain bioavailable and also, to develop new drugs with multi-target mechanisms in neurodegenerative disorders (i.e., tacrine-resveratrol fused hybrids) [197].

Likewise, growth factors, neuropeptides and transcriptional factors may induce the activity of endogenous antioxidants in astrocytes. For instance, VEGF and BDNF stimulate antioxidants enzyme activation such as catalase, SOD, GSH peroxidase, and neuroglobin [168]. Another example includes galanin, a highly produced neuropeptide in the CNS, which suppresses H_2_O_2_ toxicity through reduction in ERK1/2 pathway in rat cortical astrocytes [198]. On the other hand, PPAR are transcriptional factors with neuroprotective effects modulated by lipids, prostaglandins, anti-diabetic medications and non-steroidal anti-inflammatory (NSAIDs) drugs [199]. The PPAR-γ receptor agonist GL516 stimulates catalase activity, reducing ROS production and apoptosis in the CTX-TNA2 rat astrocyte cell line [200]. Regarding PPAR activity and oxidative stress, a post-mortem study conducted on the frontal cortices from individuals with AD, nondemented individuals with AD neuropathological changes (NDAN) and healthy controls, reported increased oxidative damage and higher redox imbalance in the AD brains compared with the other groups [201]. Authors examined the astrocytes, finding a significant downregulation in the PPAR γ-coactivator 1α (PGC1α) transcription factor from the AD brains compared with the NDAN group, suggesting this difference as a possible explanation for the dissimilar response in oxidative regulation and the presence of dementia.

Finally, free radical scavengers may modify ROS in the intra and extracellular compartments [202]. In this way, some microvesicles that contain pro-oxidant and antioxidant molecules can affect ROS levels in the extracellular compartment through direct and indirect mechanisms [202]. Some scavenger molecules have been tested in in vitro models of AD and have been shown to be effective for ROS elimination. For instance, the free radical scavenger edaravone has been tested in a mouse model of AD (APP23) associated with brain chronic hypoperfusion. In such study, the protective mechanisms of edaravone include attenuation of endothelium/astrocyte unit dysfunction, and reduction in oxidative stress and neuroinflammation [203]. Other mechanisms of neuroprotection of edaravone described in animal models of AD (APPswe/PS1 mice) include inhibition of Aβ aggregation, reduction in tau hyperphosphorylation and attenuation of astrocytosis (GFAP reactivity) [204]. Anti-amyloid effects and modulation of aquaporin 4 (AQP4) expression of edaravone have been tested in models of ischemia/reperfusion injury [205]. In addition, protective effects have been shown regarding perfusion with superoxide (O_2_^−^) and hydroxyl radical (OH) scavengers (i.e., n-propyl gallate) in astrocytic primary cultures immersed in methylmercury-induced pro-oxidant microenvironment [206].

Taken together, these findings suggest that astrocyte-focused therapeutic interventions can have important protective effects reducing amyloid-induced oxidative stress through enzymatic and non-enzymatic pathways. Another neuroprotective mechanism may include an increase in the expression of genes related to antioxidant functions. Future studies should be careful to include controlled dosages and times of intervention, which are needed to address some of the previously documented limitations regarding antioxidant therapeutics. Other important factors that must be explored in future research include a specific characterization of the antioxidants used, and improved AD-specific preclinical models with amyloidosis or tauopathy. Finally, regarding the selective targeting of astrocytes, some interesting approaches include the modulation through growth factors, the suppression of H_2_O_2_ activity by intervention on the ERK1/2 pathway, and the stimulation of catalase activity by PPAR-γ receptor agonists. Interventions in the pathways implicated in both excessive ROS production and their removal could ameliorate many of the pathological landmarks of this disease.

### 3.3. Modulation of Glutamatergic Activity

As stated before, Aβ can disrupt glial function and glutamate uptake capacity with subsequent excitotoxicity. This effect may involve A2A receptors through reduction in the expression of the two major glutamate transporters in astrocytes: EAAT1 and EAAT2 [207]. FDA approved riluzole as a treatment for amyotrophic lateral sclerosis, showing it has some protective effects reducing excitotoxic pathways (decreases the presynaptic glutamate release), improving mitochondrial function and acting on lipid metabolism [208,209]. Moreover, riluzole may have astrocyte-dependent mechanisms such as enhanced GLT-1 expression in primary mouse striatal astrocytes [210]. Riluzole has been tested in an AD model (APPswe/PS1dE9 mice), showing a preventive effect on cognitive impairment induced by early life stress. The mechanism includes the increasing expression of EAAT2 transporter in the hippocampus [211].

Ceftriaxone, a third generation cephalosporin, has been shown to increase EAAT2 expression [212], with promising results in animal models of neurodegenerative disorders such as AD, Parkinson and Huntington disease [213,214,215,216,217]. Additionally, ceftriaxone has also been proven to increase the levels of several proteins implicated in glutamate uptake and metabolism. For example, it increases neuronal expression of sodium-coupled neutral amino-acid transporter 1 (SNAT1), vesicular glutamate transporters 1 and 2 (VGLUT1/2) and enhances glutaminase activity in an AD model (APP/PS1 mice). Those effects were reverted by deletion of one GLT-1 allele [218]. In addition to upregulating the expression of GLT-1, ceftriaxone may promote the glutamate-glutamine cycle (increasing glutamine synthetase and N glutamine transporter 1) and prevent cognitive impairment in AD models (APP/PS1 mice) [219]. Although this beneficial effect of ceftriaxone was not tested directly on astrocytes, it is well known that astrocytes participate in the glutamate-glutamine-GABA cycle. In a 3xTg-AD mouse model, it has been shown that ceftriaxone can reduce tau pathology and upregulate GLT-1, with the consequent attenuation of cognitive decline [213]. In the same study, a correlation was found in transgenic mice between increased GFAP reactivity and reduction in GLT-1. Moreover, it was reported a reduction in GLT-1 expression in neuron and astrocyte cocultures.

Astrocytes express functional NMDA receptors with some differences in comparison with neurons. For instance, glial NMDA receptors have low calcium permeability and lack of Mg^++^ blocking [220]. However, Aβ-induced dysfunction of glutamate receptors not only affects neurons, but may also affect astrocytic NMDA receptors, and disrupt neuron-glia transmission [221]. NMDA receptor antagonists such as MK801 and memantine, might attenuate glutamate mediated excitotoxicity in neurons and astrocytes [222]. However, pharmacological antagonism of specific NMDAR subunits (GluN2A and GluN2B) in astrocytes enhances Aβ-induced synaptotoxicity, suggesting a synaptic protective effect with the activation of GluN2 NMDA receptors. Indeed, the potential protective effects of theses subunits in astrocytes may involve a neurotrophic mediated mechanism [223].

It has been shown that Aβ was able to induce astrocytic glutamate release which led to extrasynaptic NMDA receptor activation, excitotoxicity, promote amyloid production and impair synaptic plasticity [224,225]. Therefore, selective inhibition of NMDA receptor activity may protect against Aβ-induced synaptic dysfunction in susceptible brain regions such as the hippocampus. Likewise, selective antagonists of astroglial and extrasynaptic NMDA receptors have been developed, such as UBP141 and nitromemantine, respectively. Nitromemantine inhibits calcium increase induced by Aβ, toxic NO response and reduced synaptic loss in transgenic mouse models for AD [224]. In in vitro studies, UBP141 also antagonizes NMDA astrocyte receptors, decreases calcium permeability, modifies Mg^2+^ sensitivity, and therefore, NMDA activity [226]. Levetiracetam is an anticonvulsant with multiple mechanisms of action including the modulation of glutamate release via SV2A vesicle protein, enhance GABA synthesis and increase in the expression of astrocyte glutamate transporters (EAAT1/GLAST and EAAT2/GLT-1) [227]. In vitro, levetiracetam has shown to reduce Aβ-induced glutamate release from human astrocytes presumably by interacting with astrocytic SV2A, with evidence that levetiracetam can also reduce gliotransmission-mediated excitation [228]. In a retrospective observational study conducted in patients with amnestic mild cognitive impairment or early AD, treatment with lamotrigine and levetiracetam showed both clinical benefits and good tolerability, while raising the question of the importance of future research in the role convulsions play in AD’s progression and prognosis [229]. In addition, a study in AD patients with epilepsy showed that levetiracetam was associated with better attention, short-term memory and oral fluency performance [230]. Another anticonvulsant that acts on the NMDAR that has been studied for AD is MK801, or dizolcilpine, which works as an NMDAR antagonist with analgesic and anticonvulsant properties [231]. NMDAR blocking has been successful with the FDA-approved drug memantine, but since the MK801 has a higher receptor affinity, its clinical application in AD has been limited [232].

Other possible interventions on glutamate transporters include estrogens and selective estrogen receptor modulators (SERMs), which have shown to increase both GLT-1 and GLAST via transcriptional pathways and non-genomic pathways, also activating MAPK, ERK, PI3K-Akt, TGF-α and NF-κB signaling in astrocytes. Additionally, there is evidence that estrogens can induce the release of BDNF [128]. A postmenopausal AD model with ovariectomized (OVX) female rats, identified the beneficial effect of dimethyl fumarate in both cognitive functions and astrocytic activation represented as a reduced GFAP expression [233]. The proposed mechanism to achieve the mentioned effects is the suppression of AD markers such as APOE-E1, BACE1, Aβ_1–42_, and a reduction in hyperphosphorylated tau.

Cyclin-dependent kinase 5 (CDK5), a serine-threonine kinase, has important functions regarding neuritogenesis, synapse formation and synaptic regulation [234]. It also plays important role in cognitive functions such as memory and learning, and has been implicated in AD pathophysiology, since Aβ appears to abnormally activate CDK5 secondary to p25 formation, leading to tau phosphorylation and neuronal death [235]. Experimental evidence suggests that silencing of astrocytic CDK5 protects against neurotoxicity mediated by glutamate. Roscovitine, an inhibitor of CDKs with affinity for CDK5, appears to partially reverse morphological changes produced by glutamate, including the appearance of lamellipodia and filopodia, suggesting a protective effect against excitotoxicity [236]. In the same experiment, CDK5 silencing in astrocytes reduced excitotoxicity in neurons, showing that the CDK5 shRNA-miR astrocytes exerted neuroprotection mediated by BNDF in a Rac1-dependent manner.

Taurine is an endogenously produced beta-amino acid with cytoprotective effects, due to its antioxidative and anti-inflammatory actions [237]. In the brain and retina, taurine is considered the most abundant free amino acid, although its concentrations decline with age [238,239]. Taurine predominates in astrocytes and neurons, but the latter cells require the precursor hypotaurine, released from astrocytes, in order to produce taurine [240]. Therefore, astrocytes regulate the metabolism of taurine in the CNS. Taurine possesses a similar structure to GABA, explaining why it is able to activate the GABAA and glycine receptors, and also to serve as GABAB agonist [241,242]. Furthermore, taurine helps to regulate the entry of calcium into the cell [236], and also modulates inositol trisphosphate (IP3) and intracellular calcium levels [243]. Thus, taurine plays an important homeostatic and neuroprotective role in the CNS acting as a gliotransmitter, with proposed therapeutic effects for many diseases such as epilepsy, stroke, retinal degeneration, mitochondrial diseases, and several neurodegenerative diseases including AD [244]. For instance, in chick retinal neurons treated with Aβ, taurine offers neuroprotection through both GABA activation and reduction in excitotoxicity [245]. Furthermore, taurine was shown to bind to oligomeric Aβ and recover cognitive deficits in the APP/PS1 transgenic AD mouse model [246]. More recently, taurine was reported to decrease phosphorylated tau protein levels in the cerebellum and prefrontal cortex of scopolamine-treated Wistar rats [247]. Chronic intracerebroventricular administration of taurine in the streptozotocin dementia model, improved cognitive dysfunction in Wistar rats through a regulation of cholinergic functions together with attenuation of neuroinflammation and oxidative stress [248]. In addition, taurine oral administration during 6 weeks, improved hippocampal spatial working memory tasks and decreased insoluble Aβ_1–42_ in the cortex of APP/PS1 mice [249]. According to the webpage clinicaltrials.gov (accessed on 18 October 2022), no clinical trial is currently examining the effects of taurine in dementia. However, there is a registered randomized, double-blind, placebo controlled clinical trial comparing effects of taurine supplementation on cognitive function in patients with diabetes, but unfortunately the state of the study is unknown [250]. Although not yet tested in humans with AD, taurine can be considered as a promising therapeutic candidate for AD.

Regarding possible therapeutic interventions that aim to reduce excitotoxicity, some astrocyte-focused therapeutic approaches include augmenting the astrocytic glutamate reuptake, increasing the glutamate-glutamine-GABA metabolic pathway, and selectively blocking glutamatergic receptors such as NMDA or by modulating the astrocyte-specific glutamate transporter EAAT2. Other interventions could include the indirect modulation of these mechanisms by targeting specific transcription factors and the specific regulation of gliotransmitters release. Future pharmacological approaches should take into account the multiple processes in which the astrocyte is involved in the glutamate metabolism, since reducing excitotoxicity can improve neuronal lifespan and potentially improve cognition.

### 3.4. APOE and Lipid Metabolism

There is cumulative evidence of modifications in lipid metabolism in early and late-forms of AD. These alterations include changes in synthesis, transport and cell intake of cholesterol, fatty acids, glycerophospholipids and sphingolipids [251]. Modulation in the activity of lipid transporters may have a therapeutic role in AD. For instance, it has been shown that gemfibrozil, a drug used for dyslipiaemia, combined with retinoic acid, enhances Aβ uptake in mouse primary astrocytes in a PPARα-dependent manner through the low-density lipoprotein receptor (LDLR), and precipitates degradation of Aβ through transcription factor EB (TFEB), a gene associated with autophagy and lysosomal function. Moreover, dual treatment could potentially impact astrocytic Aβ reuptake function, and Aβ plaque reduction in mice hippocampus in a 5xFAD mouse model of AD [252].

As we discussed earlier, of major importance in the pathogenesis of SAD includes APOE, a protein involved in cholesterol transport. Intracellular cholesterol levels are regulated by astrocytes through metabolic processes (i.e., via glucose-derived acetyl CoA) and cholesterol transport proteins such as APOE and through ATP-binding cassette subfamily A member 1 (ABCA1) via LXR/RXR receptors [253]. Astrocytes are the main APOE producers of the brain [254], and as explained before, APOE polymorphic alleles are possible risk or protection factors for developing AD. APOE-ε4 carriers have a higher risk of developing SAD compared with those carrying APOE-ε3, while APOE-ε2 carriers are thought to have the lowest risk [59]. The risk of having AD by APOE-ε4 includes complex interactions with amyloid metabolism, increase in pro-inflammatory processes and a decrease in anti-inflammatory factors. In addition, APOE-ε4 is related to arginine increment by microglia, thus increasing ROS [255].

Interest in APOE-based therapeutics arises by the number of mechanisms this molecule can interact in AD pathophysiological processes including neuroinflammation, amyloid burden, cerebrovascular integrity and synaptic plasticity [256]. In an interesting and unusual clinical case, it was reported a patient that, despite carrying a *PSN1* mutation with high levels of amyloid in the brain, did not experience cognitive decline until her seventies (three decades after the expected onset of cognitive decline). This patient had two copies of the APOE-ε3 Christchurch (R136S) mutation (ApoE3ch) which modulates the Aβ aggregation, thus reducing the generation of fibrillary forms of Aβ_1–42_ [257]. Such findings are compatible with the above-mentioned role of APOE-ε3 as a protective factor against AD development. Likewise, Lin et al., used CRISPR/Cas9 gene editing to generate APOE-ε4 induced pluripotent stem cells (iPSCs) from parental APOE-ε3 cells in order to obtain neurons, astrocytes and microglia-like cells with AD phenotypes [258]. APOE-ε4 neurons showed increased synapse number and Aβ_1–42_ release, while astrocytes displayed impaired Aβ uptake and elevated intracellular levels of cholesterol. Some of those pathological effects (except for Aβ_1–42_ aggregates) were reverted after genetic modification from APOE-ε4 to APOE-ε3. Considering the role of different APOE isoforms in AD development, new therapeutic strategies have been tested focusing on APOE-ε4 as a target: reduction in APOE levels, targeting APOE-ε4 protein and indirect mechanisms which regulate APOE receptors [259]. Moreover, transgenic mice (P301S tau/Aldh1l1-Cre/apoE3) with deletion of APOE-ε4 showed a reduction in astrocyte activation, decreased phosphorylated tau accumulation, attenuation of brain atrophy and reduction in microglial synaptic pruning [66]. Likewise, anti-human APOE antibody (HAE-4) in 5xFAD mice model reduced Aβ deposition, amyloid angiopathy and also dampened reactive microglial, astrocytic, and proinflammatory-associated genes in the cortex [260]. Interaction between APOE-ε4 and Aβ is related to less amyloid clearance due to unstable complexes formation. Specific blocking of the binding site of APOE on Aβ with a synthetic peptide (Aβ_12–28_P) reduce amyloid deposition and reduces tau pathology [261,262].

Another strategy includes retinoid (RXR) and liver (LXR) receptor agonists which increase APOE lipidation in astrocytes ABCA1, a transporter particularly involved in the efflux of molecules from the brain [259]. LXR are expressed in astrocytes and have functions such as regulating immune cell response and cholesterol metabolism. An in vitro study demonstrated that treatment with the LXR agonist TO901317 in astrocytes, increases Aβ clearance thought interactions with microglial cells and APOE lipidation [263]. Other studies have shown that RXR (i.e., bexarotene) and LXR agonists (i.e., GW3965) reduce astrogliosis, decrease proinflammatory cytokines and promote several anti-amyloid mechanisms [264,265].

Since APOE is involved in nearly all of the pathophysiological mechanisms of AD, it is important for future research to focus on specific interventions regarding its expression, associated metabolic pathways and modulation of its receptors. Increasing APOE lipidation and blocking the APOE-Aβ interactions have been shown to reduce many of the pathological alterations evidenced in AD, including astrogliosis and neuroinflammation. Interestingly, intervention focused on the PPARα and lipid transporters appears to improve degradation of Aβ plaques. Future interventions could attempt to design drugs that can specifically target astrocytic mechanisms involved in lipid metabolism pathways.

### 3.5. AGE Inhibitors

Advanced glycation-end products (AGE) are considered to play a role in the generation of ROS, vascular inflammation, cellular apoptosis and gene expression alterations that may contribute to AD [266]. AGE accumulates gradually during the aging process, interfering with normal protein glycation, folding and enhancing abnormal protein aggregation [267]. In addition, AGE´s are increased in altered metabolic states as happens in metabolic syndrome and in dementia, and these pathological conditions have been associated with the presence of dementia [268]. Moreover, there is evidence of up-regulation of the RAGE receptor in AD models [269], an effect that involves both neurons and astrocytes [270]. In turn, RAGE is activated in the astrocyte by Aβ to induce neuroinflammation, oxidative stress and excitotoxicity [93,271]. Azeliragon (TTP488), a RAGE inhibitor with anti-inflammatory effects, was proposed as a potential AD therapeutic agent, however, its clinical trial was terminated at phase III due to lack of efficiency (NCT02080364). Although, in a recent study, it has been demonstrated the inhibitory activity on RAGE/SERT of 34 different azeliragon-vilazodone chimeric compounds in neuroblastoma-derived cells, of which only one shown a reduction in Aβ_25–35_-induced cytotoxicity. However, the neuroprotective effects of dual inhibitors RAGE/SERT remains to be confirmed in an astrocytic model of AD [272]. Azeliragon has shown to reverse amyloid (Aβ_1–42_) and neuroinflammatory (induced by LPS) injury via the JAK-STAT pathway, and to reduce NLRP1-mediated inflammasome activation [273]. Azeliragon administration significantly reduced neuronal damage in an in vivo AD rat model, and co-administered with the JAK inhibitor tofacitinib and the STAT inhibitor fludarabine, showed further effects in AD reversion [274]. However, future studies are needed in order to assess the azeliragon beneficial mechanisms in neurodegenerative disorders.

The coupling of RAGE with the NF-κB pathway represents a very important signal in the production of pro-inflammatory agents. The presence of Aβ has been shown to activate the RAGE receptor and induce a pro-inflammatory profile in astrocytes. Furthermore, the presence of AGE´s due to excessive glycated proteins, as occurs in metabolic syndrome/diabetes, also activates RAGE receptors. As neuroinflammation is a critical component of AD, interventions envisioned to attenuate the Aβ-induced proinflammatory state can prove helpful.

### 3.6. Neurovascular Unit and Blood–Brain Barrier Interventions

Astrocytes are an important structural and functional component of the NVU and the BBB [275,276]. In the BBB, astrocytes help to regulate metabolic requirements of neural cells through glucose uptake and oxygen delivery based on the energetic needs. Functionally, these cells play a vasoactive role in the BBB by mediating vasodilation by epoxyeicosatrienoic acids (EET) and PGE2, and vasoconstriction by 20-hydroxyeicosatetraenoic acid [277]. There is evidence suggesting that the integrity of the NVU and BBB is affected in all neurodegenerative disorders to some degree, including mild cognitive impairment and AD [278,279,280]. Vascular dysfunction is now being recognized as a prominent feature in prodromal AD, and functional changes in cerebral blood flow (CBF) have been shown to be associated with the rate of accumulation of Aβ in the brain. BBB integrity and permeability can be altered in dementia-associated disorders such as brain traumatic injury, vascular alterations and neuroinflammation [281].

Early BBB alterations have been evidenced in both grey and white matter at early stages of AD, and there is an important overlap of both cerebrovascular pathologies in its late-onset and autosomal dominant forms [282]. Astrocytic degeneration and cellular atrophy impair the structural integrity of the BBB, with a dysfunction of the NVU following these cellular changes induced by AD. Atrophic astrocytes have been found in post-mortem brain tissue of AD patients and are strongly implicated in a reduced coverage of the brain blood vessels by the astrocytic endfeet. This contributes to a dysfunction in the NVU, including vascular deficits observed in early stages of the disease [277]. Chronic neuroinflammation induced by astrocytes may affect NVU function. For example, a study with post-mortem human samples of AD patients evidenced that vessel distribution was found altered in the gyrus frontalis medius and hippocampus, with a positive correlation of RA with Braak stages in the cortex suggesting that the increased GFAP immunoreactivity found within the NVU could reflect RA associated with small vessels. This could trigger vascular dysfunction and extravasation of peripheral immune cells into the brain, thus perpetuating neuroinflammation [283]. Furthermore, there is evidence that extracellular vesicles (EV), a form of cellular communication that can transfer molecules, organelles and metabolites to other cells, are implicated in the pathophysiological processes of AD since they are able to mediate leukocyte infiltration in neuroinflammatory processes, and alter neuronal branching and firing [284]. An experimental study conducted by Gonzalez-Medina and collaborators evidenced that astrocyte-derived EV (AEV) induced astrocyte reactivity, with neuroglial cytotoxic effects and gap widening in the endothelium found specifically in familial AD (FAD), as well as endothelial alterations mainly found in SAD. Interestingly, it also showed that neurons treated with culture medium from adult 3xTg-AD astrocytes showed decreased viability and morphological alterations. These findings suggest that AEV, in both forms of the disease, are capable of causing endothelial disruption and astrocyte hyperreactivity surrounding the BBB [285].

Some authors have suggested that CDK5 inhibition could be a potential target for AD, not only by its role in reverting cellular alterations following glutamate excitotoxicity, but also by its role in NVU integrity. CDK5 inhibition appears to have a protective effect on endothelial cells in models of ischemia and hypoxia, with both in vivo and in vitro evidence of intracellular gap reversion, restoration of proteins implicated in adhesion, and an important effect in NVU integrity. In addition, CDK5 silencing appears to prevent endothelial activation during immune cell infiltration in the CNS. These processes appear to be mediated by astrocytic-BDNF release, which has a protective function that promotes neuronal survival and is associated with the CREB pathway, which is related to cognitive deficit recovery. These findings suggest that CDK5 inhibition is a promising therapeutic target in AD that needs further research in specific experimental models of the disease [236].

Interestingly, APOE-ε4 is involved in the brain’s microvascular integrity. An experimental study conducted by Bell and authors showed that APOE-ε4 expression can induce BBB breakdown via the CypA–NF-κB–matrix-metalloproteinase-9 (MMP-9) pathway in pericytes of both APOE-deficient and APOE-ε4-expressing mice [286]. Interestingly, astrocyte-secreted APOE-ε3 suppressed this pathway through a lipoprotein receptor, and TR-APOE-ε3, TR-APOE-ε2 and murine APOE maintained the structural integrity of the BBB. These findings suggest an important role of APOE-ε4 in promoting BBB alterations that could lead to neurodegenerative changes, and a possible promising future astrocytic therapeutic target based on APOE modulation.

Taken together, these findings suggest that therapeutic interventions which prevent NVU alterations and BBB damage, could delay or even avert the progression of AD. An important therapeutic approach is CDK5 inhibition, since it appears to have restorative effects in endothelial cells and stimulates both astrocytic-BDNF and the CREB pathway. However, since these findings have been only described in ischemia models, further studies including specific AD models are suggested. Another intervention includes the modulation of astrocytic APOE, since APOE-ε4 appears to be involved in BBB breakdown, while other isoforms appear to have protective effects in murine models.

### 3.7. Interventions on Glymphatic System

The glymphatic system model explains part of the CSF-interstitial fluid (ISF) efflux and clearance of metabolic products from the CNS to the periphery mainly through AQP4, a protein expressed in astrocyte endfeet processes. Both AQP4 inhibitors and *AQP4* gene deletion appear to slow down clearance of Aβ, APOE, tau and SOD1 [287]. In AD, Aβ drainage appears to involve the glymphatic system to some degree, since Aβ has been detected in cervical lymph nodes in murine models of the disease [21]. During both sleep and with the administration of certain anesthetics that induce slow-wave sleep, the interstitial space is increased, which results in decreased resistance to fluid flow and therefore increased interstitial solute clearance [288,289,290]. A relationship between AD and sleep disorders is well established, where the glymphatic system may have a main role in their pathophysiology [291]. Correspondingly, AQP4 expression and its localization in astrocytic endfeet are affected by sleep deprivation, and therefore, may contribute to reduction in Aβ and tau clearance [292]. In humans, a post-mortem AD case series observed that perivascular localization of AQP4 is affected, and its expression is reduced in frontal grey matter associated with increased Aβ and neurofibrillary pathological burden [293]. Similarly, a study with APP/PS1 mice and AQP4 knockout, concluded that AQP4 deletion severely impairs glymphatic clearance function, decreasing Aβ clearance but without Aβ plaque deposition [294]. Some strategies seek to restore or improve the clearance function through the modification of the glymphatic system in order to delay or prevent the onset of AD. Indeed, in a rodent study with transgenic mice reported that n-3 polyunsaturated fatty acids could promote interstitial Aβ clearance through an increase in AQP4 function [295]. In addition, different studies have investigated whether the induction of AQP4 expression during AD might contribute to improving disease course. However, it has been shown that AQP4 overexpression occurs in inadequate positions away from astrocyte endfeet where its presence does not contribute to the removal of waste products [296,297]. Furthermore, other molecules such as L-3-n-butylphthalide have been shown to increase Aβ clearance by enhancing perivascular AQP4 localization [298].

Since the glymphatic system plays an important role in the clearance of substances in the CNS, interventions focused on astrocytes can improve the removal of Aβ. Because inhibition of AQP4 expressed in astrocyte endfeet clearly affects its functions, future interventions could promote Aβ clearance by improving AQP4 function, such as n-3 polyunsaturated fatty acid supplementation. Future research needs to take into account the fact that the expression of AQP4 must be targeted at the astrocytic endfeet, since overexpression of AQP4 in other sites appears to be ineffective. Taken together, these findings suggest that astrocytes could play a pivotal role in AD therapeutics as research on the glymphatic system continues to evidence a significant role in the disease. Due to the critical relationship between the glymphatic system and sleep, strategies involving the promotion of sleep hygiene complemented pharmacological interventions may be of benefit for individuals with AD.

### 3.8. Aβ Clearance

Dysfunction of Aβ clearance and its consequent accumulation has been characterized as a contributing factor to AD. The low-density lipoprotein receptor-related protein1 (LRP1) has been described as part of the astrocytic and neuronal Aβ clearance [299]. In fact, astrocytes and neurons adjacent to amyloid plaques have shown an augmented expression of LRP1 compared with other brain cells [300]. Multiple drugs have shown to enhance Aβ clearance by increasing LRP1 expression, such as pioglitazone, a PPARγ agonist [301]. In addition, pioglitazone was used in a rat model demonstrating a significant increase in LRP1 expression with consequent reduction in Aβ deposits and Aβ_1–42_ levels [301].

Furthermore, neprilysin (NEP) also contributes to Aβ clearance, showing an inverse relationship with Aβ accumulation [302]. Apelt and colleagues [303], demonstrated an upregulated astrocytic expression of NEP in the Tg2576 mice AD model. Therefore, the enhancement of NEP expression, as occurs with the administration of drugs like somatostatin or somatostatin receptor agonists, provide a potential therapeutic target for AD as previously proven in primary neuronal cells [304]. However, its effects in an astrocytic AD model remains to be confirmed. On the other hand, a study with cultured rat astrocytes treated with epigallocatechin gallate, a phenolic antioxidant, resulted in enhanced NEP release into the extracellular space, and in exogenous Aβ degradation through activation of the ERK-PI3K pathway [305].

Metalloproteinases, more specifically MMP-2 and MMP-9, released by astrocytes, play an important role in Aβ clearance [53]. A study evaluated iPSC-derived astrocytes from both SAD and FAD patients, demonstrating increased GFAP expression and MMP production, and contributing to an increased Aβ clearance after exposure to monomeric and aggregated tau [306]. Sirtuin 1 (SIRT1), a NAD^+^-dependent protein deacetylase highly conserved in mammals, has been previously shown to reduce Aβ load in neurons, with an inhibition of glial inflammatory response. In astrocytes, SIRT1 appears to promote intracellular Aβ degradation by deacetylase activity and lysosomal pathways. This clearance was higher in oligomeric Aβ than in fibrillar forms, suggesting that astrocytes could be more involved in Aβ clearance during the initial stages of the disease [307].

NB-02, a standardized mix derived from plant-based extracts of *Poria cocos* and *Morus alba* L., previously known as DA-9803, has been studied as a potential AD therapeutic intervention. A study with APP/PS1 mice showed that NB-02 halted amyloid plaque deposition and preserved neuronal calcium homeostasis [308]. Moreover, in the same study it was shown that NB-02 also restored spine density and transformed the morphology of astrocytes as well as microglia to a more phagocytic state. Interestingly, similar results were reported in 5xFAD mice [309]. Both authors suggest that NB-02 could be acting by decreasing Aβ oligomerization and production while increasing clearance, but the exact mechanism of action is still not understood.

The accumulation of Aβ is one of the pathological hallmarks of AD. Although Aβ on its own cannot explain all the molecular and cellular alterations observed in AD, an excessive presence of undesired Aβ forms, such as Aβ_1–42_, compromises the function of all brain cells. Therefore, improving the clearance and preventing the aggregation of Aβ can be considered as an important therapeutic target. This strategy will most likely involve astrocytes, as these cells express many enzymes such as neprilysin, insulin degrading-enzyme, and several MMP´s, involved in Aβ degradation. In addition, the glymphatic system, dependent on astrocytes, has been recently pointed as the main CNS waste clearance system, promoting the elimination of proteins including Aβ peptides.

### 3.9. Calcium Signalling

Astrocytic calcium signaling has shown important effects on behavior, cognition and emotion according to several models [310]. Most astrocytic functions, including the regulation of synaptic activity, require intracellular calcium elevations in single cells and on vast glial networks [311]. These transient calcium elevations are needed in order to modulate the synaptic response by gliotransmission [312]. Calcium currents can be either of spontaneous origin or mediated by neuronal activity by cholinergic and glutamatergic receptors [310,313]. The calcium required for astrocyte functions can be obtained from various sources including the extracellular medium (via transient receptor potentials or voltage calcium channels), or from intracellular compartments such as mitochondrial storage [314], and from the endoplasmic reticulum via the IP3 pathway [315]. Regarding the cellular calcium flux in astrocytes, it appears to be mediated by the transient receptor potential A1 (TRPA1) channels, which are also involved in GABA transport by sodium- and chloride-dependent GABA transporter 3 (GAT-3), and in the regulation of astrocyte resting calcium levels [316]. In addition, the TRPA1 channels appear to have a role in hippocampal LTP by mediating the calcium needed for the activation of the NMDA receptor [316]. Regarding AD, calcium dysregulation has been reported in neurons in cognitive decline associated with the aging process, with a downregulation of calcium clearance processes being a consistent finding across studies [317]. Moreover, a synergic mechanism between calcium dysregulation and Aβ deposits could aggravate neurodegenerative processes and cognitive decline in AD patients [318]. Since intracellular calcium is needed for the astrocytic release of glutamate [319], a possible role of calcium dysregulation as a source of astrocytic-mediated glutamate excitotoxicity needs to be taken into account.

The calcium signaling sensing receptor (CaSR), expressed in both astrocytes and neurons, is a member of family C G-protein coupled receptors that interacts with changes in extracellular calcium levels, and is involved in multiple signaling pathways [320]. There is evidence suggesting that in human astrocytes, CaSR interacts with Aβ plaques. Aβ aggregates appear to promote downregulation of this receptor, and as a consequence, neurons appear to secrete free radicals and de novo Aβ [321]. As a promising therapeutic agent, NPS 2143 effectively blocks the Aβ-induced CaSR signaling, suppressing endogenous Aβ_1–42_ secretion by human cortical astrocytes and HCN-1A neurons induced by fibrillar Aβ_25–35_ [322], with an important suppression of intracellular accumulation of p-tau and its exosomal release via the same pathway [323]. In addition, NPS 2143 appears to suppress the release of the proinflammatory mediators IL-6, regulated upon activation, normal T cell expressed and presumably secreted (RANTES), soluble intercellular adhesion molecule 1 (ICAM-1) and monocyte chemotactic protein-2 (MCP-2) in normal adult human astrocytes [324].

TRP canonical 6 (TRPC6), a non-selective cationic channel expressed in astrocytes with a plethora of functions in the CNS, has been implicated in the pathophysiology of AD and other pathological processes including neuroinflammation and ischemia [325]. Hyperforin, a TRPC6 agonist, can increase intracellular calcium levels, reduce astrogliosis and disaggregate Aβ aggregates, with effects on spatial memory [326,327]. Hyperforin appears to reduce mushroom spine loss in both APP knock-in and presenilin mouse models by stimulating the nSOC pathway formed by the TRPC6 and the Orai2 channels [328]. There is experimental evidence suggesting that hyperforin can reduce both tau phosphorylation and Aβ_1–42_ production in pheochromocytoma-derived PC12 cells by regulation of the Akt-GSK3β signaling pathway [329]. Further studies could assess if a similar mechanism underlies its effects on astrocytes. Interestingly, tetrahydrohyperforin (IDN5706), a semi-synthetic derivative of hyperforin, appears to decrease large Aβ deposits, reduce oxidative damage and prevent RA inflammatory response, alleviating memory decline evidenced during behavioral testing in APPswe and PS-1dE9 mouse models of AD [330]. In a double transgenic APPswe/PSEN1ΔE9 model of AD, tetrahydroperforin decreased proteolytic processing of APP, reduced tau hyperphosphorylation and astrogliosis [331]. Tetrahydroperforin modulates Aβ production by interacting with APP and C99, blocking the cleavage of C99 by the γ-secretase [326].

The endocannabinoid pathway in astrocytes appears to involve the regulation of intracellular calcium levels. Both cannabinoid receptors 1 (CB1) and 2 (CB2) are expressed in astrocytes [332], suggesting that endocannabinoids and cannabinoid agonists may be interesting astrocytic therapeutic approaches in AD. CB1 is expressed in physiological conditions [333,334], while CB2 appears to be expressed as a result of Aβ-derived neuroinflammation [335]. Regarding possible therapeutic agents, WIN 55,212-2, a synthetic cannabinoid that acts as an agonist of both CB1 and CB2, increased antioxidant agents and the anti-inflammatory response in rat astrocytes exposed to Aβ_1–42_ [336]. Interestingly, the phytocannabinoid cannabidiol (CBD) appears to reduce GFAP, IL-1β and iNOS expression in mice exposed to Aβ_1–42_ [337]. Some authors suggest that, regarding CBD treatment in AD models, both the observed decrease in reactive gliosis and in neurodegeneration are dependent on PPARγ pathway activation (PMID: 22163051), which is also involved in calcium signalling [338].

The purinergic P2Y1 receptor (P2Y1R) has a prominent role in glial calcium signalling and AD pathology, including Aβ metabolism, neuroinflammation and neurovascular coupling [339,340]. There is evidence of enhanced calcium signalling in mouse models of AD where P2Y signalling is associated with the release of ATP from RA [341]. Chronic treatment with MRS2179, an P2Y1R antagonist, returned hyperactive astrocytes to control levels and normalized astroglial network activity, diminished neuronal network dysfunction and improved synaptic plasticity in APP/PS1 mice [342]. These protective effects are independent of amyloid burden, but the modified morphological changes of RA were observed close to senile plaques. However, the same authors established that the beneficial effects of MRS2179 are not dependent on signalling pathways downstream of P2Y1R in astrocytes.

Cellular calcium regulation is affected by many pathophysiological processes involved in AD, including Aβ deposits, neuroinflammation and tau. In turn, the resultant calcium dysregulation in both neurons and astrocytes appears to have important effects on behavioral alterations and cognitive decline related to the natural course of the disease. Further studies are needed in order to better elucidate the causal relationship between calcium dysregulation and AD. However, it should be noted that these calcium-acting therapeutic approaches appear to reduce and reverse many of the pathological processes of the disease, including RA response and astrogliosis, reduction in oxidative damage, decrease in proinflammatory cytokine release, reduction in intracellular accumulation and hyperphosphorylation of tau, and disaggregation of Aβ plaques. Interestingly, since calcium dysregulation is also involved in abnormal glutamate astrocytic release, interventions in these pathways could be an indirect form of reducing glutamate-mediated excitotoxicity. These findings suggest that astrocytic calcium regulation can be an important therapeutic option that can have an impact in many of the pathological changes of the disease, and that it should be explored in future experiments.

### 3.10. Melatonin-Based Interventions

Decreased levels of melatonin have been found in cerebrospinal fluid of AD patients, which continue to decline as the disease progresses [343]. Thus, melatonin supplementation has proven useful in the circadian rhythm regulation, contributing to cognitive function improvement [344]. In contrast, inhibition of melatonin synthesis increases tau phosphorylation and Aβ toxicity [345]. To exemplify, Zhang and colleagues [346] studied a rat model injected with Aβ_1–42_ intracerebroventricularly 24 h after melatonin administration, demonstrating improved synaptic function, attenuation of astrogliosis and enhanced spatial memory. The same study identified a significant decrease in GFAP expression in neuron-astrocyte co-culture injected with both Aβ_1–42_ and melatonin. The proposed mechanism for which this is obtained is through the inhibition of Notch1 signalling pathway [346]. Moreover, melatonin has shown to attenuate kainic acid-induced astrocyte responses determined by immunohistochemical detection of GFAP, possibly by antioxidant and anti-inflammatory actions [347].

Out of the three subtypes of melatonin receptors, MT1 and MT2 predominate in astrocytes and microglia compared to neurons [348]. Multiple signalling pathways depend on the activation of such receptors, including a disintegrin and metalloproteinase domain-containing protein 10 (ADAM10), BACE1 and GSK-3 which decrease Aβ synthesis and enhance Aβ clearance [349]. Ramelteon, a highly selective MT1 and MT2 agonist, has been proposed as a potential therapeutic agent in AD [350]. However, no studies were found regarding its specific action in an astrocytic AD model. Furthermore, Xiang and colleagues [351], evaluated melatonin-induced APOE expression in mouse astrocytes, demonstrating an increase in APOE expression as a result of the activation of PI3K/eNOS signalling pathways.

Melatonin appears to modulate many of the pathophysiological alterations of AD, including neuroinflammation and Aβ clearance, and it may also play a role in improving cognitive functions such as spatial memory and sleep. This makes it an interesting therapeutic target with multiple mechanisms of action. Future pharmaceutical approaches could include the specific targeting of MT1 and MT2 in astrocyte models, alongside other interventions on the Notch-1 pathway and kainic-acid astrocytic responses. However, even with the aforementioned effects on astrogliosis and astrocyte reactivity, more evidence is needed in order to explore the possibilities of astrocyte-centered melatonin interventions.

## 4. Non-Pharmacological Interventions That Impact Astrocyte Function

There are several non-pharmacological treatments that have been studied in AD such as physical and social stimulation, diet and memory training. In a recent meta-analysis, the potential effect of acupuncture, exercise intervention, music, cognitive therapy, and repetitive transcranial magnetic stimulation (TCMS) was described, but with very low quality to moderate quality of evidence [352]. As follows, we will describe the impact of some of these AD therapies in the astrocytic function.

### 4.1. Physical Exercise

Physical exercise is associated with an improvement in cognitive function and as a preventive intervention in AD [353,354]. Regarding astrocytes and physical exercise, a recent study investigated the effect of voluntary exercise on astrocyte modulation in mice, with increased GFAP expression being reported as a neuromodulatory response to exercise in AD [355]. Furthermore, there were morphological changes in hippocampal astrocytes, which consisted of increased soma size and atrophied branches in plaque-associated RA, alongside the restoration of reduced BDNF level in GFAP-positive astrocytes. This suggests LTP stabilization associated with reduction in cognitive decline [356].

Most of the research into physical activity in AD has focused on the general benefits of exercise in dementia patients. However, when taken together, the aforementioned findings show interesting effects in the astrocyte, including effects on synaptic plasticity and cognitive reserve. Future studies could explore the relationship of physical activity with lactate production, since lactate metabolism in astrocytes appears to be involved in cognitive processes such as learning and has been associated with the improved mood in people that exercise regularly.

### 4.2. Dietary Approaches: Ketogenic Diet

Regarding dietary approaches, a potential role for implementing a ketogenic diet in AD has been proposed, taking into account the bioenergetic disruption that precipitates multiple neurological diseases. A recent study in mice showed that implementation of an in vivo ketogenic diet increased gene expression of neuronal molecular pathways while simultaneously suppressing their astrocytic counterparts [357]. More specifically, this study emphasizes AD-related genes, such as *Psen1*, *Aph1*, *Bace1*, and *Apoe*, which were upregulated in neurons, and simultaneously downregulated in astrocytes, as demonstrated by relative mRNA expression. Regarding clinical evidence, a case report of a 59-year-old patient with mild AD and type 2 diabetes showed that a 10-week lifestyle intervention that included a ketogenic diet, with adherence monitored by weekly blood ketone levels, showed a symptomatic improvement in AD Montreal cognitive assessments, with a 30% improvement in its score. Additionally, the patient’s memory improved, since his ability to name animals post-intervention showed a 74% improvement compared to its pre-intervention scores. This is by a presumed restoration of mitochondrial function secondary to ketone body production and the monocarboxylate transporter pathway [358]. In a recent randomized crossover trial, ketogenic diet applied in 21 AD patients, showed improvements in daily function and quality of life, and possible a reduction in cardiovascular risk factors [359].

Further studies are needed in order to recommend a ketogenic dietary approach to AD patients. However, the differences between neuronal and astrocytic responses to high-fat, low-carbohydrate diets show an interesting area for future research. Furthermore, the interaction between high fat diet and specific APOE isoforms could be explored in order to better characterize how ketonic bodies interact with altered lipid metabolism according to the APOE alterations, and also have an individualized dietary approach.

### 4.3. Electromagnetic and Electric Stimulation

Electromagnetic and electric stimulation are non-pharmacological interventions in AD [360], with important effects documented on both human and murine astrocytes. For example, in an AD animal model (5xFAD mice), repetitive TCMS increase the drainage efficiency of glymphatic system and meningeal lymphatics, thus reducing both Aβ deposits and astrogliosis [361]. A 28% and 10% reduction in Aβ-induced ROS production after exposure to electromagnetic fields (EMF) in rat and human primary astrocytes, respectively, has been shown [362]. In the same study, the administration of EMF was shown to ameliorate depolarization of the mitochondrial membrane in rat primary astrocytes exposed to Aβ_1–42_ and staurosporine, a protein kinase inhibitor. In fact, there is evidence of the beneficial effects of TCMS in AD patients, from mild [363], to moderate and severe grades of disease progression [364]. However, it is not clear if the mechanisms of the beneficial effect of TCMS in AD involve astrocytes function directly.

The beneficial effects of electroacupuncture have been tested extensively in animal models of AD, and include several mechanisms of action such as neuroprotective (through anti-inflammatory and antioxidative effects), regulation of metabolism and neurotrophic factor release, and reducing amyloid deposition [365]. In a recent study in SAMP8 mice showed that electroacupuncture therapy reduced Aβ accumulation, inhibited astrocyte reactivity, and improved AQP4 polarity [366]. In a postoperative cognitive dysfunction model in rats, it was shown that electroacupuncture may reverse cognitive impairment reducing GFAP-positive astrocyte number in the hippocampus, modulates oxidative stress and increase glial cell-derived neurotrophic factors [367]. However, the therapeutic effect of acupuncture in patients with vascular dementia and AD has shown limited efficacy [368,369].

Summarizing, these findings suggest that electro-magnetic stimulation might have important effects regarding oxidative stress, mitochondrial dysfunction and clearance of Aβ. Many specific astrocytic targets appear to respond to these interventions, including AQP4, GFAP reactivity and the release of both ROS and many neurotrophic factors. Further studies may explore these interventions in specific AD models, since it appears that electromagnetic and electric stimulation seems to have transversal effects in nearly all the pathophysiological processes involved in the disease.

**Figure 1:**
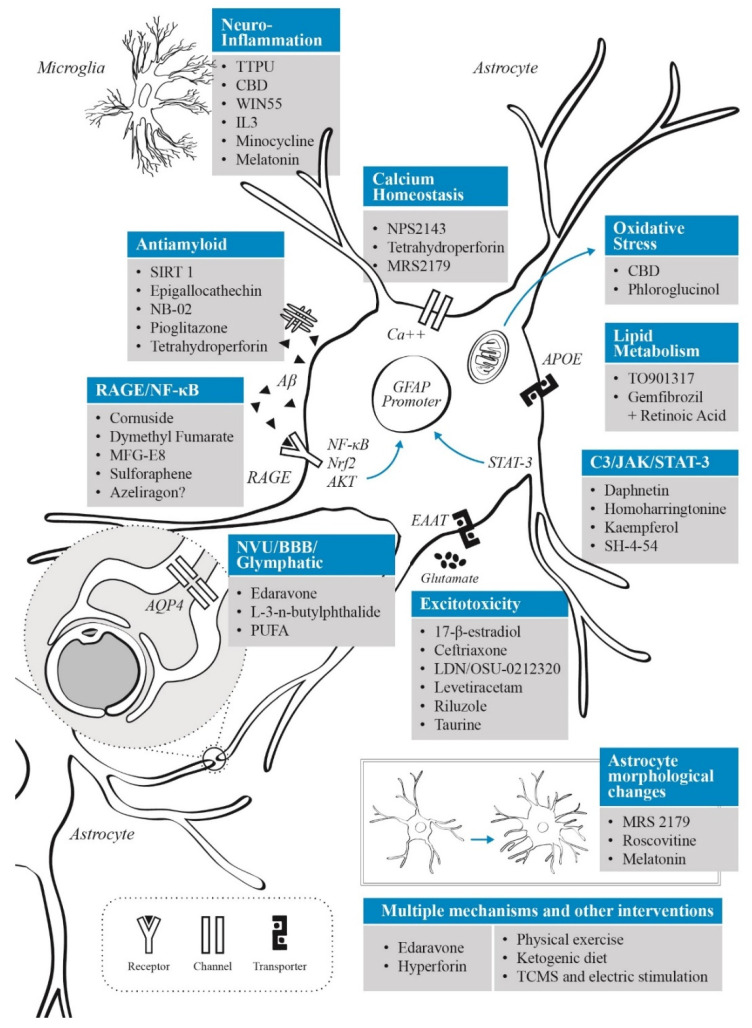
Schematic representation of the main pathophysiological astrocytic-based mechanisms in AD. Therapies associated with their respective targets are illustrated. Most of the molecules described here have several action mechanisms (including reduced GFAP expression and glial reactivity), however, they have a main target in the astrocyte. Those molecules whose main action mechanism involve major morphological changes were described separately. Abbreviations: apolipoprotein E (APOE); aquaporin 4 (AQP4); blood–brain barrier (BBB); cannabidiol (CBD); excitatory amino acid transporters (EAAT); neurovascular unit (NVU); n-3 polyunsaturated fatty acids (PUFA); receptor for advanced glycation end products (RAGE); sirtuin 1 (SIRT 1); signal transducer and activator of transcription 3 (STAT-3): transcranial magnetic stimulation (TCMS); 1-trifluoromethoxyphenyl-3-urea (TPPU).

A summary of the pharmacological and molecular interventions that have an effect on astrocytes and was probed in AD models is presented in Table 1 and Figure 1.

## 5. Conclusions

Considering the limitations of the existing AD clinical studies and preclinical models, new approaches have to be implemented. In this regard, astrocyte-focused therapies need to be considered as an important alternative to current AD treatments. These should include interventions aimed to modify neuroinflammation, improve lipid metabolism (alongside with APOE function), and enhance glymphatic system function, among others. Astrocytes must be considered as a key agent in future AD therapeutic approaches due to their crucial role in CNS homeostatic control and the evidenced participation in several aspects of the disease. New targets and mechanisms reviewed here include the up-regulation of EAAT2 or GLT-1 expression, modulation of RAGE-NF-κB and Notch1 signaling pathways, inhibition of the C3/STAT3 pathways, the regulation of the PPARα-dependent pathway, an augmented release of astrocytic IL-3, the up-regulation of SOCS3, the astrocytic SV2A, an indirect modulation of the microglia response, and other astrocyte-specific factors. Since AD is a multifactorial disease, combined therapies with different mechanisms of action (for instance, an inhibitor of RAGE/NF-κB pathway together with a ROS scavenger) may enhance the therapeutic effects of astrocytes in early stages of AD. Additional characteristics of astrocyte-based therapies include numerous effects involving not only glial, but also neuronal and vascular function, together with the modulation of tau phosphorylation and amyloid metabolism. Potential future therapies based on the regulation of astrocyte function could include selective modification of specific gene expression through viral vectors with certain tropism to astrocytes, nanoparticle-delivered astrocytic molecules that cross BBB, extracellular vesicles directed to the SNC that improve astrocyte homeostasis, hybrid drugs with integrated action mechanisms, cell replacement therapy (i.e., graft of astrocytes with a neuroprotective profile), perfusion with glia-conditioned medium derived from astrocytes and molecular induction of astrocytes in a physiological state which may reverse or halt pathological changes in AD.

## Figures and Tables

**Table 1:** Astrocytic therapeutic targets and molecules used in preclinical studies of AD. In the present table only molecules and drugs (in alphabetic order) that were probed in AD models (i.e., amyloidosis, tauopathy or transgenic animals) and also, that modulate directly or indirectly the astrocyte activity, were included.

Reference	Therapeutic Agent	Astrocytic Target and Mechanism	AD Model	Therapeutic Effects
[155]	1-trifluoromethoxyphenyl-3-(1-propionylpiperidin-4-yl) urea (TPPU)	Blocks astroglial sEH up-regulation, suppression of microglial reactivity through astrocyte-microglia	5xFAD mice	Reduction in proinflammatory gene expression, reversion of immune pathways dysregulation, reduction in gliosis, reduction in number and size of Aβ plaques, cognitive improvement (object recognition test and fear conditioning paradigm)
[370]	17-β-estradiol	Increases astrocytic glutamate uptake by an unknown mechanism that appears to be independent of estrogen receptors Increases expression of GLT-1 and GLAST in astrocytes	AD Human astrocytes	Increment glutamate reuptake in astrocytes
[337]	Cannabidiol (CBD)	Reduces GFAP, IL-1β and iNOS expression in mice exposed to Aβ_1–42_ Reduces Aβ-generated reactive gliosis	3–5-month-old C57BL/6J mice	Reduces neuroinflammation. Delays onset and progression of Aβ neurotoxicity
[213,219]	Ceftriaxone	IncreaseGLT-1 expression, glutamine synthetase and N glutamine transporter 1	APP/PS1 mice 3xTg-AD mouse model	Decreases cognitive impairment. Promotes the glutamate-glutamine cycle. Reduces tau pathology
[167]	Cornuside	AKT/Nrf2/NF-κB pathway and reduction in proinflammatory cytokines	3xTg-AD mice	Prevention of cognitive impairment. Anti-amyloid. Reduction in tau phosphorylation Anti-inflammatory (reducing IL-1β, IL-6, TNF-α levels) Antioxidant
[137]	Daphnetin	Inhibits STAT3 phosphorylation at Ser727. Decreases astrogliosis (GFAP expression)	APP/PS1 mice	Reduces area and amount of Aβ deposition, decreases the soluble Aβ_1–40_ and Aβ_1–42_.
[233]	Dymethyl fumarate	Reduction in GFAP reactivity. Reduction in NF-κB-mediated inflammatory response. Activation of AMPK/SIRT-1 & AKT/CREB/BDNF hubs	D-galactose (D-Gal) administered to ovariectomized (OVX) female rats (postmenopausal AD model)	Ameliorated memory deficits. Anti-inflammatory. Antioxidant effects (via SOD and GSH). Reduction in tauo-/amyloidopathy
[203,204]	Edaravone	Attenuation of endothelium/astrocyte unit dysfunction. Reduction in astrocytosis (GFAP reactivity)	APP23 rodents associated with brain chronic hypoperfusion APPswe/PS1 mice	Reduction in oxidative stress and neuroinflammation. Improved damaged myelin (enhancing oligodendrocytes). Inhibition of Aβ aggregation and reduction in tau hyperphosphorylation
[305]	Epigallocatechin gallate	Increased expression of NEP	Cultured rat astrocytes treated with EGCG	Increased degradation of exogenous Aß
[252]	Gemfibrozil + Retinoic acid	PPARα-dependent pathway, low-density lipoprotein receptor, transcription factor EB	5xFAD mice	RA switched to a neuroprotective state, lowered Aβ in brain, increased lysosomal astrocytic activity, increased autophagic flux, improved spatial learning and memory, enhancement in cognitive function, and reduction in Aβ plaques in hippocampus
[371]	Homoharringtonine	STAT3 signalling in glial cells Increases SOCS3 expression in the hippocampus.	APP/PS1 Mice	Alleviates cognitive deficits. Reduces accumulation of Aβ_1–40_ and Aβ_1–42_ in both soluble and insoluble forms. Attenuates synaptic function impairment. Suppresses STAT3 activation and reduces neuroinflammation
[328,329]	Hyperforin	TRPC6 agonist that stimulates activity of the nSOC pathway in the spines Regulates the Akt-GSK3β signaling pathway	APP knock-in and presenilin mouse models	Increases intracellular calcium levels, reduces astrogliosis, disaggregates Aβ aggregates, rescues mushroom spine loss Reduces tau phosphorylation and Aβ_1–42_ production in PC12 cells
[96]	IL-3	Astrocytic IL-3 induces microglia activation and instructs it to clear aggregates of Aβ and tau.	5xFAD mice	Allows microglia to clear aggregates of Aβ and tau. Reduced memory decline and Aβ load
[140,141]	Kaempferol	Complement C3 and STAT 3 pathways	AD model induced by ICV streptozotocin in Wistar rats	Prevents the activation of complement C3 protein and the generation of neurotoxic astrocytes. Antiamiloid, antioxidative (increased SOD and GSH levels) and anti-inflammatory mechanisms
[298]	L-3-n-butylphthalide	AQP4	APP/PS1 mice	Reduce Aβ deposition and enhance perivascular localization of AQP4.
[129]	LDN/OSU-0212320	Increases EAAT2 function	APPswe mice Primary neuron and astrocyte mixed culture	Reduce cognitive impairment and amyloid burden. Prevent Aβ_25–35_ oligomer–induced toxicity
[228]	Levetiracetam	Astrocytic SV2A	Primary cultures of human astrocytes exposed with oligomeric Aβ_1–42_	Reduction in Aβ-induced glutamate release, reduction in gliotransmission-mediated excitation
[346]	Melatonin	Inhibition of Notch1 signalling pathway	Mice ICV injection with Aβ_1–42_	Improved synaptic function, attenuation of astrogliosis and enhanced spatial memory performance, decrease in GFAP expression
[173,174]	MFG-E8	Downregulation of NF-κB and upregulation of PI3K-Akt	Aβ_1–42_-activated microglia-conditioned medium to induce astrocytic activation Neuronal/glial mixed culture exposed to Aβ_1–42_	Anti-inflammatory effects on astrocytes (modulation in IL-1α, TNF, and C1q) Increase microglial endocytosis of Aβ
[147]	Minocycline	Tetracycline antibiotic-protein synthesis inhibitor and increases expression of anti-inflammatory genes	htau mouse AD model	Decrease in astrocytic and caspase-3 activation. Reduction in neuronal loss. Reduce the number of RA, decreases the formation of abnormal tau species and attenuated the production of proinflammatory cytokines
[342]	MRS2179	P2Y1R antagonist	Mice with human KM67/671NL mutation in APP and human L166P-mutated PS1	Increase density and branches length of astrocytic process and points of terminal process close to senile plaques. Preserve structural synaptic integrity
[295]	n-3 polyunsaturated fatty acids (PUFA)	AQP4	fat-1 mice C57BL/6 (ICV Aβ injection)	Promote interstitial Aβ clearance. Inhibit astrocyte activation and protect the AQP4 polarization
[308,309]	NB-02 (previously known as DA-9803)	Mechanism of action under study. Authors suggest that it could be acting by decreasing Aβ oligomerization and production while increasing clearance.	APP/PS1 mice, 5xFAD mice	Halts amyloid plaque deposition, preserves neuronal calcium homeostasis, restores spine density, alters astrocytic morphology, induces a phagocytic state in microglia
[322,323,324]	NPS2143 (CaSR negative allosteric modulator)	CaSR signaling secretion of proinflammatory agents Suppresses astrocytic Aβ_1–42_ by CaSR signaling.	Aβ_25–35_-exposed human cortical astrocytes Normal human adult astrocytes, fAβ_25–35_-treated astrocytes, HC-1A neurons in vitro	Reduces neuroinflammation and amyloidosis. Reduces intracellular accumulation and release of p-tau. Suppresses the release of IL-6, RANTES, ICAM-1 and MCP-2.
[189]	Phloroglucinol	Inhibits the generation of ROS	Oligomeric forms of Aβ_1–42_ in astrocyte cultures	Enhancing antioxidant enzymes expression such as SOD and GSH. Inhibits astrocytes activation induced by Aβ
[301]	Pioglitazone	Increased LRP1 expression	Senescence-accelerated mouse prone-8 (SAMP8) mice model	Reduction in Aß deposits and Aß_1–40_ levels improved performance in water maze test
[211]	Riluzole	Increase EAAT2 expression	APPswe/PS1dE9 mice	Prevent cognitive impairment and synaptic plasticity changes induced by early stress
[236]	Roscovitine	CDK inhibitor with affinity for CDK5	SAMP8 mice	Reverses morphological changes produced by glutamate excitotoxicity
[132]	SH-4-54 (STAT3 phosphorylation site inhibitor)	C3aR1-STAT3 signaling mediated astrogliosis and astrocyte reactivity	PS19 mice	Reduction in neuroinflammation and partial rescue of tau pathology
[307]	Sirtuin 1 (SIRT 1)	Promotes oligomeric Aß degradation in astrocytes by deacetylation of lysosome-related proteins and upregulation of total lysosome number	Astrocyte culture of Sprague Dawley rats cortex samples.	Reduction in Aß deposits
[159,163,165]	Sulforaphene	Inhibition of NF-κB and stimulation of Nrf2 pathways	AD-Like pathology model induced by streptozotocin and N2a/APPswe cells	Anti-inflammatory and antioxidant effects. Inhibited the phosphorylation of tau protein and improved cognitive deficit in memory function.
[246,247]	Taurine	Produced and released as gliotransmitter by astrocytes. Activates GABAA and glycine receptors, and serves as GABAB agonist	APP/PS1 transgenic AD mouse	Homeostatic and neuroprotective effects. Regulates intracellular calcium levels, binds to oligomeric Aβ and helps to decrease tau phosphorylation
[263]	TO901317	Astrocytic LXR agonist Increase ABCA1 and APOE expression in astrocytes	APP/PS1 mice	Increase phagocytosis of Aβ by microglial activity. Improve spatial learning
[326,330,331]	Tetrahydroperforin (IDN5607)	TRPC6 agonist that acts by modulating Aβ production, interacting with APP and C99, and blocking the cleavage of C99 by the γ-secretase	APPswe and PS-1dE9 mouse models	Prevents RA inflammatory response, decreases large Aβ deposits., reduces damage caused by oxidative stress, alleviates memory decline
[336]	WIN 55,212-2	CB1 and CB2 receptors	Astrocytes exposed to Aβ_1–42_	Prevented the elevation of TNF and IL-1β, p-65, COX-2 and iNOS proteins. Increased cell viability

## Data Availability

Not applicable.

## References

[1] Revi M. (2020). Alzheimer’s Disease Therapeutic Approaches. Adv. Exp. Med. Biol..

[2] Erkkinen M.G., Kim M.-O., Geschwind M.D. (2018). Clinical Neurology and Epidemiology of the Major Neurodegenerative Diseases. Cold Spring Harb. Perspect. Biol..

[3] Querfurth H.W., LaFerla F.M. (2010). Alzheimer’s Disease. N. Engl. J. Med..

[4] Ferrari C., Sorbi S. (2021). The Complexity of Alzheimer’s Disease: An Evolving Puzzle. Physiol. Rev..

[5] Diociaiuti M., Bonanni R., Cariati I., Frank C., D’Arcangelo G. (2021). Amyloid Prefibrillar Oligomers: The Surprising Commonalities in Their Structure and Activity. Int. J. Mol. Sci..

[6] Raskin J., Cummings J., Hardy J., Schuh K., Dean R.A. (2015). Neurobiology of Alzheimer’s Disease: Integrated Molecular, Physiological, Anatomical, Biomarker, and Cognitive Dimensions. Curr. Alzheimer Res..

[7] Song T., Song X., Zhu C., Patrick R., Skurla M., Santangelo I., Green M., Harper D., Ren B., Forester B.P. (2021). Mitochondrial Dysfunction, Oxidative Stress, Neuroinflammation, and Metabolic Alterations in the Progression of Alzheimer’s Disease: A Meta-Analysis of in Vivo Magnetic Resonance Spectroscopy Studies. Ageing Res. Rev..

[8] Armstrong R.A. (2019). Risk Factors for Alzheimer’s Disease. Folia Neuropathol..

[9] Knopman D.S., Amieva H., Petersen R.C., Chételat G., Holtzman D.M., Hyman B.T., Nixon R.A., Jones D.T. (2021). Alzheimer Disease. Nat. Rev. Dis. Prim..

[10] Liu C.-C., Liu C.-C., Kanekiyo T., Xu H., Bu G. (2013). Apolipoprotein E and Alzheimer Disease: Risk, Mechanisms and Therapy. Nat. Rev. Neurol..

[11] Kabir M.T., Uddin M.S., Setu J.R., Ashraf G.M., Bin-Jumah M.N., Abdel-Daim M.M. (2020). Exploring the Role of PSEN Mutations in the Pathogenesis of Alzheimer’s Disease. Neurotox. Res..

[12] Verkhratsky A., Nedergaard M. (2018). Physiology of Astroglia. Physiol. Rev..

[13] Beard E., Lengacher S., Dias S., Magistretti P.J., Finsterwald C. (2021). Astrocytes as Key Regulators of Brain Energy Metabolism: New Therapeutic Perspectives. Front. Physiol..

[14] Verkhratsky A., Nedergaard M., Hertz L. (2015). Why Are Astrocytes Important?. Neurochem. Res..

[15] Murat C.D.B., García-Cáceres C. (2021). Astrocyte Gliotransmission in the Regulation of Systemic Metabolism. Metabolites.

[16] Santello M., Toni N., Volterra A. (2019). Astrocyte Function from Information Processing to Cognition and Cognitive Impairment. Nat. Neurosci..

[17] Bélanger M., Magistretti P.J. (2009). The Role of Astroglia in Neuroprotection. Dialogues Clin. Neurosci..

[18] Liu B., Teschemacher A.G., Kasparov S. (2017). Neuroprotective Potential of Astroglia. J. Neurosci. Res..

[19] Valori C.F., Guidotti G., Brambilla L., Rossi D. (2019). Astrocytes: Emerging Therapeutic Targets in Neurological Disorders. Trends Mol. Med..

[20] McConnell H.L., Mishra A. (2022). Cells of the Blood-Brain Barrier: An Overview of the Neurovascular Unit in Health and Disease. Methods Mol. Biol..

[21] Louveau A., Plog B.A., Antila S., Alitalo K., Nedergaard M., Kipnis J. (2017). Understanding the Functions and Relationships of the Glymphatic System and Meningeal Lymphatics. J. Clin. Investig..

[22] Garwood C.J., Ratcliffe L.E., Simpson J.E., Heath P.R., Ince P.G., Wharton S.B. (2017). Review: Astrocytes in Alzheimer’s Disease and Other Age-Associated Dementias: A Supporting Player with a Central Role. Neuropathol. Appl. Neurobiol..

[23] Arranz A.M., De Strooper B. (2019). The role of astroglia in Alzheimer's disease: Pathophysiology and clinical implications. Lancet Neurol..

[24] González-Reyes R.E., Nava-Mesa M.O., Vargas-Sánchez K., Ariza-Salamanca D., Mora-Muñoz L. (2017). Involvement of Astrocytes in Alzheimer’s Disease from a Neuroinflammatory and Oxidative Stress Perspective. Front. Mol. Neurosci..

[25] Chun H., Lee C.J. (2018). Reactive Astrocytes in Alzheimer’s Disease: A Double-Edged Sword. Neurosci. Res..

[26] Frost G.R., Li Y.-M. (2017). The Role of Astrocytes in Amyloid Production and Alzheimer’s Disease. Open Biol..

[27] Ibrahim A.M., Pottoo F.H., Dahiya E.S., Khan F.A., Kumar J.B.S. (2020). Neuron-Glia Interactions: Molecular Basis of Alzheimer’s Disease and Applications of Neuroproteomics. Eur. J. Neurosci..

[28] Nanclares C., Baraibar A.M., Araque A., Kofuji P. (2021). Dysregulation of Astrocyte-Neuronal Communication in Alzheimer’s Disease. Int. J. Mol. Sci..

[29] Acosta C., Anderson H.D., Anderson C.M. (2017). Astrocyte Dysfunction in Alzheimer Disease. J. Neurosci. Res..

[30] Monterey M.D., Wei H., Wu X., Wu J.Q. (2021). The Many Faces of Astrocytes in Alzheimer’s Disease. Front. Neurol..

[31] Wyss-Coray T., Loike J.D., Brionne T.C., Lu E., Anankov R., Yan F., Silverstein S.C., Husemann J. (2003). Adult Mouse Astrocytes Degrade Amyloid-Beta in Vitro and in Situ. Nat. Med..

[32] Pihlaja R., Koistinaho J., Malm T., Sikkilä H., Vainio S., Koistinaho M. (2008). Transplanted Astrocytes Internalize Deposited Beta-Amyloid Peptides in a Transgenic Mouse Model of Alzheimer’s Disease. Glia.

[33] Singh D. (2022). Astrocytic and Microglial Cells as the Modulators of Neuroinflammation in Alzheimer’s Disease. J. Neuroinflamm..

[34] Saroja S.R., Gorbachev K., Julia T., Goate A.M., Pereira A.C. (2022). Astrocyte-Secreted Glypican-4 Drives APOE4-Dependent Tau Hyperphosphorylation. Proc. Natl. Acad. Sci. USA.

[35] Carter S.F., Herholz K., Rosa-Neto P., Pellerin L., Nordberg A., Zimmer E.R. (2019). Astrocyte Biomarkers in Alzheimer’s Disease. Trends Mol. Med..

[36] Fakhoury M. (2018). Microglia and Astrocytes in Alzheimer’s Disease: Implications for Therapy. Curr. Neuropharmacol..

[37] Uddin M.S., Lim L.W. (2022). Glial Cells in Alzheimer’s Disease: From Neuropathological Changes to Therapeutic Implications. Ageing Res. Rev..

[38] Valenza M., Facchinetti R., Menegoni G., Steardo L., Scuderi C. (2021). Alternative Targets to Fight Alzheimer’s Disease: Focus on Astrocytes. Biomolecules.

[39] Pekny M., Nilsson M. (2005). Astrocyte Activation and Reactive Gliosis. Glia.

[40] Escartin C., Galea E., Lakatos A., O’Callaghan J.P., Petzold G.C., Serrano-Pozo A., Steinhäuser C., Volterra A., Carmignoto G., Agarwal A. (2021). Reactive Astrocyte Nomenclature, Definitions, and Future Directions. Nat. Neurosci..

[41] Heneka M.T., Carson M.J., El Khoury J., Landreth G.E., Brosseron F., Feinstein D.L., Jacobs A.H., Wyss-Coray T., Vitorica J., Ransohoff R.M. (2015). Neuroinflammation in Alzheimer’s Disease. Lancet Neurol..

[42] Liddelow S.A., Guttenplan K.A., Clarke L.E., Bennett F.C., Bohlen C.J., Schirmer L., Bennett M.L., Münch A.E., Chung W.-S., Peterson T.C. (2017). Neurotoxic Reactive Astrocytes Are Induced by Activated Microglia. Nature.

[43] Calsolaro V., Matthews P.M., Donat C.K., Livingston N.R., Femminella G.D., Guedes S.S., Myers J., Fan Z., Tyacke R.J., Venkataraman A.V. (2021). Astrocyte Reactivity with Late-Onset Cognitive Impairment Assessed in Vivo Using 11C-BU99008 PET and Its Relationship with Amyloid Load. Mol. Psychiatry.

[44] Diaz-Amarilla P., Arredondo F., Dapueto R., Boix V., Carvalho D., Santi M.D., Vasilskis E., Mesquita-Ribeiro R., Dajas-Bailador F., Abin-Carriquiry J.A. (2022). Isolation and Characterization of Neurotoxic Astrocytes Derived from Adult Triple Transgenic Alzheimer’s Disease Mice. Neurochem. Int..

[45] Shah D., Gsell W., Wahis J., Luckett E.S., Jamoulle T., Vermaercke B., Preman P., Moechars D., Hendrickx V., Jaspers T. (2022). Astrocyte Calcium Dysfunction Causes Early Network Hyperactivity in Alzheimer’s Disease. Cell Rep..

[46] Ferrari-Souza J.P., Ferreira P.C.L., Bellaver B., Tissot C., Wang Y.-T., Leffa D.T., Brum W.S., Benedet A.L., Ashton N.J., De Bastiani M.A. (2022). Astrocyte Biomarker Signatures of Amyloid-β and Tau Pathologies in Alzheimer’s Disease. Mol. Psychiatry.

[47] Pillai A.G., Nadkarni S. (2022). Amyloid Pathology Disrupts Gliotransmitter Release in Astrocytes. PLoS Comput. Biol..

[48] Andersen J.V., Christensen S.K., Westi E.W., Diaz-delCastillo M., Tanila H., Schousboe A., Aldana B.I., Waagepetersen H.S. (2021). Deficient Astrocyte Metabolism Impairs Glutamine Synthesis and Neurotransmitter Homeostasis in a Mouse Model of Alzheimer’s Disease. Neurobiol. Dis..

[49] Ong W.-Y., Tanaka K., Dawe G.S., Ittner L.M., Farooqui A.A. (2013). Slow Excitotoxicity in Alzheimer’s Disease. J. Alzheimer’s Dis..

[50] Selkoe D.J., Hardy J. (2016). The Amyloid Hypothesis of Alzheimer’s Disease at 25 Years. EMBO Mol. Med..

[51] Kametani F., Hasegawa M. (2018). Reconsideration of Amyloid Hypothesis and Tau Hypothesis in Alzheimer’s Disease. Front. Neurosci..

[52] Karran E., De Strooper B. (2022). The Amyloid Hypothesis in Alzheimer Disease: New Insights from New Therapeutics. Nat. Rev. Drug. Discov..

[53] Yin K.-J., Cirrito J.R., Yan P., Hu X., Xiao Q., Pan X., Bateman R., Song H., Hsu F.-F., Turk J. (2006). Matrix Metalloproteinases Expressed by Astrocytes Mediate Extracellular Amyloid-Beta Peptide Catabolism. J. Neurosci..

[54] Montoliu-Gaya L., Mulder S.D., Veerhuis R., Villegas S. (2017). Effects of an Aβ-Antibody Fragment on Aβ Aggregation and Astrocytic Uptake Are Modulated by Apolipoprotein E and J Mimetic Peptides. PLoS ONE.

[55] Liu C.-C., Hu J., Zhao N., Wang J., Wang N., Cirrito J.R., Kanekiyo T., Holtzman D.M., Bu G. (2017). Astrocytic LRP1 Mediates Brain Aβ Clearance and Impacts Amyloid Deposition. J. Neurosci..

[56] Heneka M.T., Sastre M., Dumitrescu-Ozimek L., Dewachter I., Walter J., Klockgether T., Van Leuven F. (2005). Focal Glial Activation Coincides with Increased BACE1 Activation and Precedes Amyloid Plaque Deposition in APP[V717I] Transgenic Mice. J. Neuroinflamm..

[57] Zhao J., O’Connor T., Vassar R. (2011). The Contribution of Activated Astrocytes to Aβ Production: Implications for Alzheimer’s Disease Pathogenesis. J. Neuroinflamm..

[58] Huang Y., Mahley R.W. (2014). Apolipoprotein E: Structure and Function in Lipid Metabolism, Neurobiology, and Alzheimer’s Diseases. Neurobiol. Dis..

[59] Liao F., Yoon H., Kim J. (2017). Apolipoprotein E Metabolism and Functions in Brain and Its Role in Alzheimer’s Disease. Curr. Opin. Lipidol..

[60] Lanfranco M.F., Sepulveda J., Kopetsky G., Rebeck G.W. (2021). Expression and Secretion of ApoE Isoforms in Astrocytes and Microglia during Inflammation. Glia.

[61] Arnaud L., Benech P., Greetham L., Stephan D., Jimenez A., Jullien N., García-González L., Tsvetkov P.O., Devred F., Sancho-Martinez I. (2022). APOE4 Drives Inflammation in Human Astrocytes via TAGLN3 Repression and NF-ΚB Activation. Cell Rep..

[62] Liu C.-C., Zhao J., Fu Y., Inoue Y., Ren Y., Chen Y., Doss S.V., Shue F., Jeevaratnam S., Bastea L. (2022). Peripheral ApoE4 Enhances Alzheimer’s Pathology and Impairs Cognition by Compromising Cerebrovascular Function. Nat. Neurosci..

[63] Ba M., Kong M., Li X., Ng K.P., Rosa-Neto P., Gauthier S. (2016). Is ApoE ε 4 a Good Biomarker for Amyloid Pathology in Late Onset Alzheimer’s Disease?. Transl. Neurodegener..

[64] Wisniewski T., Drummond E. (2020). APOE-Amyloid Interaction: Therapeutic Targets. Neurobiol. Dis..

[65] Carter D.B. (2005). The Interaction of Amyloid-Beta with ApoE. Subcell Biochem..

[66] Wang C., Xiong M., Gratuze M., Bao X., Shi Y., Andhey P.S., Manis M., Schroeder C., Yin Z., Madore C. (2021). Selective Removal of Astrocytic APOE4 Strongly Protects against Tau-Mediated Neurodegeneration and Decreases Synaptic Phagocytosis by Microglia. Neuron.

[67] Wang P., Ye Y. (2021). Astrocytes in Neurodegenerative Diseases: A Perspective from Tauopathy and α-Synucleinopathy. Life.

[68] Lebouvier T., Pasquier F., Buée L. (2017). Update on Tauopathies. Curr. Opin. Neurol..

[69] Šimić G., Babić Leko M., Wray S., Harrington C., Delalle I., Jovanov-Milošević N., Bažadona D., Buée L., de Silva R., Di Giovanni G. (2016). Tau Protein Hyperphosphorylation and Aggregation in Alzheimer’s Disease and Other Tauopathies, and Possible Neuroprotective Strategies. Biomolecules.

[70] Kahlson M.A., Colodner K.J. (2015). Glial Tau Pathology in Tauopathies: Functional Consequences. J. Exp. Neurosci..

[71] Ferrer I., López-González I., Carmona M., Arregui L., Dalfó E., Torrejón-Escribano B., Diehl R., Kovacs G.G. (2014). Glial and Neuronal Tau Pathology in Tauopathies: Characterization of Disease-Specific Phenotypes and Tau Pathology Progression. J. Neuropathol. Exp. Neurol..

[72] Leyns C.E.G., Holtzman D.M. (2017). Glial Contributions to Neurodegeneration in Tauopathies. Mol. Neurodegener..

[73] Amro Z., Yool A.J., Collins-Praino L.E. (2021). The Potential Role of Glial Cells in Driving the Prion-like Transcellular Propagation of Tau in Tauopathies. Brain Behav. Immun. Health.

[74] Smith A.M., Davey K., Tsartsalis S., Khozoie C., Fancy N., Tang S.S., Liaptsi E., Weinert M., McGarry A., Muirhead R.C.J. (2022). Diverse Human Astrocyte and Microglial Transcriptional Responses to Alzheimer’s Pathology. Acta Neuropathol..

[75] Yuste-Checa P., Trinkaus V.A., Riera-Tur I., Imamoglu R., Schaller T.F., Wang H., Dudanova I., Hipp M.S., Bracher A., Hartl F.U. (2021). The Extracellular Chaperone Clusterin Enhances Tau Aggregate Seeding in a Cellular Model. Nat. Commun..

[76] Lambert J.-C., Heath S., Even G., Campion D., Sleegers K., Hiltunen M., Combarros O., Zelenika D., Bullido M.J., Tavernier B. (2009). Genome-Wide Association Study Identifies Variants at CLU and CR1 Associated with Alzheimer’s Disease. Nat. Genet..

[77] Harrington A.J., Raissi A., Rajkovich K., Berto S., Kumar J., Molinaro G., Raduazzo J., Guo Y., Loerwald K., Konopka G. (2016). MEF2C Regulates Cortical Inhibitory and Excitatory Synapses and Behaviors Relevant to Neurodevelopmental Disorders. eLife.

[78] Adrião A., Santana I., Ribeiro C., Cancela M.L., Conceição N., Grazina M. (2022). Identification of a Novel Mutation in MEF2C Gene in an Atypical Patient with Frontotemporal Lobar Degeneration. Neurol. Sci..

[79] Beecham G.W., Hamilton K., Naj A.C., Martin E.R., Huentelman M., Myers A.J., Corneveaux J.J., Hardy J., Vonsattel J.-P., Younkin S.G. (2014). Genome-Wide Association Meta-Analysis of Neuropathologic Features of Alzheimer’s Disease and Related Dementias. PLoS Genet..

[80] Lambert J.C., Ibrahim-Verbaas C.A., Harold D., Naj A.C., Sims R., Bellenguez C., DeStafano A.L., Bis J.C., Beecham G.W., Grenier-Boley B. (2013). Meta-Analysis of 74,046 Individuals Identifies 11 New Susceptibility Loci for Alzheimer’s Disease. Nat. Genet..

[81] Wang X., Lopez O.L., Sweet R.A., Becker J.T., DeKosky S.T., Barmada M.M., Demirci F.Y., Kamboh M.I. (2015). Genetic Determinants of Disease Progression in Alzheimer’s Disease. J. Alzheimer’s Dis..

[82] Wang H., Devadoss D., Nair M., Chand H.S., Lakshmana M.K. (2022). Novel Alzheimer Risk Factor IQ Motif Containing Protein K Is Abundantly Expressed in the Brain and Is Markedly Increased in Patients with Alzheimer’s Disease. Front. Cell Neurosci..

[83] Kunkle B.W., Grenier-Boley B., Sims R., Bis J.C., Damotte V., Naj A.C., Boland A., Vronskaya M., van der Lee S.J., Amlie-Wolf A. (2019). Genetic Meta-Analysis of Diagnosed Alzheimer’s Disease Identifies New Risk Loci and Implicates Aβ, Tau, Immunity and Lipid Processing. Nat. Genet..

[84] Li Q.S., De Muynck L. (2021). Differentially Expressed Genes in Alzheimer’s Disease Highlighting the Roles of Microglia Genes Including OLR1 and Astrocyte Gene CDK2AP1. Brain Behav. Immun. Health.

[85] Wang P., Ye Y. (2021). Filamentous Recombinant Human Tau Activates Primary Astrocytes via an Integrin Receptor Complex. Nat. Commun..

[86] Silva I., Silva J., Ferreira R., Trigo D. (2021). Glymphatic System, AQP4, and Their Implications in Alzheimer’s Disease. Neurol. Res. Pract..

[87] Richetin K., Steullet P., Pachoud M., Perbet R., Parietti E., Maheswaran M., Eddarkaoui S., Bégard S., Pythoud C., Rey M. (2020). Tau Accumulation in Astrocytes of the Dentate Gyrus Induces Neuronal Dysfunction and Memory Deficits in Alzheimer’s Disease. Nat. Neurosci..

[88] Piacentini R., Li Puma D.D., Mainardi M., Lazzarino G., Tavazzi B., Arancio O., Grassi C. (2017). Reduced Gliotransmitter Release from Astrocytes Mediates Tau-Induced Synaptic Dysfunction in Cultured Hippocampal Neurons. Glia.

[89] Rostami J., Holmqvist S., Lindström V., Sigvardson J., Westermark G.T., Ingelsson M., Bergström J., Roybon L., Erlandsson A. (2017). Human Astrocytes Transfer Aggregated Alpha-Synuclein via Tunneling Nanotubes. J. Neurosci..

[90] Zaheer S., Thangavel R., Sahu S.K., Zaheer A. (2011). Augmented Expression of Glia Maturation Factor in Alzheimer’s Disease. Neuroscience.

[91] Gimsa U., Mitchison N.A., Brunner-Weinzierl M.C. (2013). Immune Privilege as an Intrinsic CNS Property: Astrocytes Protect the CNS against T-Cell-Mediated Neuroinflammation. Mediat. Inflamm..

[92] Guerriero F., Sgarlata C., Francis M., Maurizi N., Faragli A., Perna S., Rondanelli M., Rollone M., Ricevuti G. (2017). Neuroinflammation, Immune System and Alzheimer Disease: Searching for the Missing Link. Aging Clin. Exp. Res..

[93] Gonzalez-Reyes R.E., Rubiano M.G. (2018). Astrocyte´s RAGE: More Than Just a Question of Mood. Cent. Nerv. Syst. Agents Med. Chem..

[94] Elangovan S., Holsinger R.M.D. (2020). Cyclical Amyloid Beta-Astrocyte Activity Induces Oxidative Stress in Alzheimer’s Disease. Biochimie.

[95] Liu W., Tang Y., Feng J. (2011). Cross Talk between Activation of Microglia and Astrocytes in Pathological Conditions in the Central Nervous System. Life Sci..

[96] McAlpine C.S., Park J., Griciuc A., Kim E., Choi S.H., Iwamoto Y., Kiss M.G., Christie K.A., Vinegoni C., Poller W.C. (2021). Astrocytic Interleukin-3 Programs Microglia and Limits Alzheimer’s Disease. Nature.

[97] Britschgi M., Rufibach K., Huang S.L.B., Clark C.M., Kaye J.A., Li G., Peskind E.R., Quinn J.F., Galasko D.R., Wyss-Coray T. (2011). Modeling of Pathological Traits in Alzheimer’s Disease Based on Systemic Extracellular Signaling Proteome. Mol. Cell Proteom..

[98] Kiddle S.J., Thambisetty M., Simmons A., Riddoch-Contreras J., Hye A., Westman E., Pike I., Ward M., Johnston C., Lupton M.K. (2012). Plasma Based Markers of [11C] PiB-PET Brain Amyloid Burden. PLoS ONE.

[99] Soares H.D., Potter W.Z., Pickering E., Kuhn M., Immermann F.W., Shera D.M., Ferm M., Dean R.A., Simon A.J., Swenson F. (2012). Plasma Biomarkers Associated with the Apolipoprotein E Genotype and Alzheimer Disease. Arch. Neurol..

[100] Di Benedetto G., Burgaletto C., Bellanca C.M., Munafò A., Bernardini R., Cantarella G. (2022). Role of Microglia and Astrocytes in Alzheimer’s Disease: From Neuroinflammation to Ca^2+^ Homeostasis Dysregulation. Cells.

[101] Sama D.M., Norris C.M. (2013). Calcium Dysregulation and Neuroinflammation: Discrete and Integrated Mechanisms for Age-Related Synaptic Dysfunction. Ageing Res. Rev..

[102] Sies H., Berndt C., Jones D.P. (2017). Oxidative Stress. Annu. Rev. Biochem..

[103] Gella A., Durany N. (2009). Oxidative Stress in Alzheimer Disease. Cell Adhes. Migr..

[104] Islam M.T. (2017). Oxidative Stress and Mitochondrial Dysfunction-Linked Neurodegenerative Disorders. Neurol. Res..

[105] Kropf E., Fahnestock M. (2021). Effects of Reactive Oxygen and Nitrogen Species on TrkA Expression and Signalling: Implications for ProNGF in Aging and Alzheimer’s Disease. Cells.

[106] Dringen R., Pfeiffer B., Hamprecht B. (1999). Synthesis of the Antioxidant Glutathione in Neurons: Supply by Astrocytes of CysGly as Precursor for Neuronal Glutathione. J. Neurosci..

[107] Wang X.F., Cynader M.S. (2000). Astrocytes Provide Cysteine to Neurons by Releasing Glutathione. J. Neurochem..

[108] Ye B., Shen H., Zhang J., Zhu Y.-G., Ransom B.R., Chen X.-C., Ye Z.-C. (2015). Dual Pathways Mediate β-Amyloid Stimulated Glutathione Release from Astrocytes. Glia.

[109] Garg S.K., Vitvitsky V., Albin R., Banerjee R. (2011). Astrocytic Redox Remodeling by Amyloid Beta Peptide. Antioxid. Redox Signal..

[110] Zoufal V., Mairinger S., Krohn M., Wanek T., Filip T., Sauberer M., Stanek J., Kuntner C., Pahnke J., Langer O. (2020). Measurement of Cerebral ABCC1 Transport Activity in Wild-Type and APP/PS1-21 Mice with Positron Emission Tomography. J. Cereb. Blood Flow Metab..

[111] Allaman I., Gavillet M., Bélanger M., Laroche T., Viertl D., Lashuel H.A., Magistretti P.J. (2010). Amyloid-Beta Aggregates Cause Alterations of Astrocytic Metabolic Phenotype: Impact on Neuronal Viability. J. Neurosci..

[112] Akama K.T., Van Eldik L.J. (2000). Beta-Amyloid Stimulation of Inducible Nitric-Oxide Synthase in Astrocytes Is Interleukin-1beta- and Tumor Necrosis Factor-Alpha (TNFalpha)-Dependent, and Involves a TNFalpha Receptor-Associated Factor- and NFkappaB-Inducing Kinase-Dependent Signaling Mechanism. J. Biol. Chem..

[113] Dallérac G., Rouach N. (2016). Astrocytes as New Targets to Improve Cognitive Functions. Prog. Neurobiol..

[114] Huffels C.F.M., Middeldorp J., Hol E.M. (2022). Aß Pathology and Neuron-Glia Interactions: A Synaptocentric View. Neurochem. Res..

[115] Nava-Mesa M.O., Jiménez-Díaz L., Yajeya J., Navarro-Lopez J.D. (2014). GABAergic Neurotransmission and New Strategies of Neuromodulation to Compensate Synaptic Dysfunction in Early Stages of Alzheimer’s Disease. Front. Cell Neurosci..

[116] Garaschuk O., Verkhratsky A. (2019). GABAergic Astrocytes in Alzheimer’s Disease. Aging.

[117] Jo S., Yarishkin O., Hwang Y.J., Chun Y.E., Park M., Woo D.H., Bae J.Y., Kim T., Lee J., Chun H. (2014). GABA from Reactive Astrocytes Impairs Memory in Mouse Models of Alzheimer’s Disease. Nat. Med..

[118] Lee M., Schwab C., McGeer P.L. (2011). Astrocytes Are GABAergic Cells That Modulate Microglial Activity. Glia.

[119] Andersen J.V., Schousboe A., Verkhratsky A. (2022). Astrocyte Energy and Neurotransmitter Metabolism in Alzheimer’s Disease: Integration of the Glutamate/GABA-Glutamine Cycle. Prog. Neurobiol..

[120] Alfaro-Ruiz R., Martín-Belmonte A., Aguado C., Hernández F., Moreno-Martínez A.E., Ávila J., Luján R. (2021). The Expression and Localisation of G-Protein-Coupled Inwardly Rectifying Potassium (GIRK) Channels Is Differentially Altered in the Hippocampus of Two Mouse Models of Alzheimer’s Disease. Int. J. Mol. Sci..

[121] Jeremic D., Sanchez-Rodriguez I., Jimenez-Diaz L., Navarro-Lopez J.D. (2021). Therapeutic Potential of Targeting G Protein-Gated Inwardly Rectifying Potassium (GIRK) Channels in the Central Nervous System. Pharmacol. Ther..

[122] Sánchez-Rodríguez I., Djebari S., Temprano-Carazo S., Vega-Avelaira D., Jiménez-Herrera R., Iborra-Lázaro G., Yajeya J., Jiménez-Díaz L., Navarro-López J.D. (2020). Hippocampal Long-Term Synaptic Depression and Memory Deficits Induced in Early Amyloidopathy Are Prevented by Enhancing G-Protein-Gated Inwardly Rectifying Potassium Channel Activity. J. Neurochem..

[123] Djebari S., Iborra-Lázaro G., Temprano-Carazo S., Sánchez-Rodríguez I., Nava-Mesa M.O., Múnera A., Gruart A., Delgado-García J.M., Jiménez-Díaz L., Navarro-López J.D. (2021). G-Protein-Gated Inwardly Rectifying Potassium (Kir3/GIRK) Channels Govern Synaptic Plasticity That Supports Hippocampal-Dependent Cognitive Functions in Male Mice. J. Neurosci..

[124] Hubbard J., Binder D.K. (2016). Astrocytes and Epilepsy.

[125] Danysz W., Parsons C.G. (2003). The NMDA Receptor Antagonist Memantine as a Symptomatological and Neuroprotective Treatment for Alzheimer’s Disease: Preclinical Evidence. Int. J. Geriatr. Psychiatry.

[126] Rothstein J.D., Dykes-Hoberg M., Pardo C.A., Bristol L.A., Jin L., Kuncl R.W., Kanai Y., Hediger M.A., Wang Y., Schielke J.P. (1996). Knockout of Glutamate Transporters Reveals a Major Role for Astroglial Transport in Excitotoxicity and Clearance of Glutamate. Neuron.

[127] Mahmoud S., Gharagozloo M., Simard C., Gris D. (2019). Astrocytes Maintain Glutamate Homeostasis in the CNS by Controlling the Balance between Glutamate Uptake and Release. Cells.

[128] Pajarillo E., Rizor A., Lee J., Aschner M., Lee E. (2019). The Role of Astrocytic Glutamate Transporters GLT-1 and GLAST in Neurological Disorders: Potential Targets for Neurotherapeutics. Neuropharmacology.

[129] Takahashi K., Kong Q., Lin Y., Stouffer N., Schulte D.A., Lai L., Liu Q., Chang L.-C., Dominguez S., Xing X. (2015). Restored Glial Glutamate Transporter EAAT2 Function as a Potential Therapeutic Approach for Alzheimer’s Disease. J. Exp. Med..

[130] Terao I., Honyashiki M., Inoue T. (2022). Comparative efficacy of lithium and aducanumab for cognitive decline in patients with mild cognitive impairment or Alzheimer’s disease: A systematic review and network meta-analysis. Ageing Res. Rev..

[131] Prillaman M. (2022). Alzheimer’s Drug Slows Mental Decline in Trial—But Is It a Breakthrough?. Nature.

[132] Litvinchuk A., Wan Y.-W., Swartzlander D.B., Chen F., Cole A., Propson N.E., Wang Q., Zhang B., Liu Z., Zheng H. (2018). Complement C3aR Inactivation Attenuates Tau Pathology and Reverses an Immune Network Deregulated in Tauopathy Models and Alzheimer’s Disease. Neuron.

[133] Wu T., Dejanovic B., Gandham V.D., Gogineni A., Edmonds R., Schauer S., Srinivasan K., Huntley M.A., Wang Y., Wang T.-M. (2019). Complement C3 Is Activated in Human AD Brain and Is Required for Neurodegeneration in Mouse Models of Amyloidosis and Tauopathy. Cell Rep..

[134] Yuan K., Ye J., Liu Z., Ren Y., He W., Xu J., He Y., Yuan Y. (2020). Complement C3 Overexpression Activates JAK2/STAT3 Pathway and Correlates with Gastric Cancer Progression. J. Exp. Clin. Cancer Res..

[135] Tsuda M., Kohro Y., Yano T., Tsujikawa T., Kitano J., Tozaki-Saitoh H., Koyanagi S., Ohdo S., Ji R.-R., Salter M.W. (2011). JAK-STAT3 Pathway Regulates Spinal Astrocyte Proliferation and Neuropathic Pain Maintenance in Rats. Brain.

[136] Toral-Rios D., Patiño-López G., Gómez-Lira G., Gutiérrez R., Becerril-Pérez F., Rosales-Córdova A., León-Contreras J.C., Hernández-Pando R., León-Rivera I., Soto-Cruz I. (2020). Activation of STAT3 Regulates Reactive Astrogliosis and Neuronal Death Induced by AβO Neurotoxicity. Int. J. Mol. Sci..

[137] Gao P., Wang Z., Lei M., Che J., Zhang S., Zhang T., Hu Y., Shi L., Cui L., Liu J. (2022). Daphnetin Ameliorates Aβ Pathogenesis via STAT3/GFAP Signaling in an APP/PS1 Double-Transgenic Mouse Model of Alzheimer’s Disease. Pharmacol. Res..

[138] Ito K., Noguchi A., Uosaki Y., Taga T., Arakawa H., Takizawa T. (2018). Gfap and Osmr Regulation by BRG1 and STAT3 via Interchromosomal Gene Clustering in Astrocytes. Mol. Biol. Cell.

[139] Reichenbach N., Delekate A., Plescher M., Schmitt F., Krauss S., Blank N., Halle A., Petzold G.C. (2019). Inhibition of Stat3-Mediated Astrogliosis Ameliorates Pathology in an Alzheimer’s Disease Model. EMBO Mol. Med..

[140] Babaei P., Eyvani K., Kouhestani S. (2021). Sex-Independent Cognition Improvement in Response to Kaempferol in the Model of Sporadic Alzheimer’s Disease. Neurochem. Res..

[141] Lopez-Sanchez C., Poejo J., Garcia-Lopez V., Salazar J., Garcia-Martinez V., Gutierrez-Merino C. (2022). Kaempferol Prevents the Activation of Complement C3 Protein and the Generation of Reactive A1 Astrocytes That Mediate Rat Brain Degeneration Induced by 3-Nitropropionic Acid. Food Chem. Toxicol..

[142] Yu L., Chen C., Wang L.-F., Kuang X., Liu K., Zhang H., Du J.-R. (2013). Neuroprotective Effect of Kaempferol Glycosides against Brain Injury and Neuroinflammation by Inhibiting the Activation of NF-ΚB and STAT3 in Transient Focal Stroke. PLoS ONE.

[143] Carow B., Rottenberg M.E. (2014). SOCS3, a Major Regulator of Infection and Inflammation. Front. Immunol..

[144] Ceyzériat K., Ben Haim L., Denizot A., Pommier D., Matos M., Guillemaud O., Palomares M.-A., Abjean L., Petit F., Gipchtein P. (2018). Modulation of Astrocyte Reactivity Improves Functional Deficits in Mouse Models of Alzheimer’s Disease. Acta Neuropathol. Commun..

[145] Chakrabarti S., Roy A., Prorok T., Patel D., Dasarathi S., Pahan K. (2019). Aspirin Up-Regulates Suppressor of Cytokine Signaling 3 in Glial Cells via PPARα. J. Neurochem..

[146] Garwood C.J., Pooler A.M., Atherton J., Hanger D.P., Noble W. (2011). Astrocytes Are Important Mediators of Aβ-Induced Neurotoxicity and Tau Phosphorylation in Primary Culture. Cell Death Dis..

[147] Garwood C.J., Cooper J.D., Hanger D.P., Noble W. (2010). Anti-Inflammatory Impact of Minocycline in a Mouse Model of Tauopathy. Front. Psychiatry.

[148] Zheng S.-Q., Gong Z.-Y., Lu C.-D., Wang P. (2017). Prostaglandin I2 Is Responsible for Ameliorating Prostaglandin E2 Stress in Stimulating the Expression of Tumor Necrosis Factor α in a β-Amyloid Protein -Dependent Mechanism. Oncotarget.

[149] Mohri I., Kadoyama K., Kanekiyo T., Sato Y., Kagitani-Shimono K., Saito Y., Suzuki K., Kudo T., Takeda M., Urade Y. (2007). Hematopoietic Prostaglandin D Synthase and DP1 Receptor Are Selectively Upregulated in Microglia and Astrocytes within Senile Plaques from Human Patients and in a Mouse Model of Alzheimer Disease. J. Neuropathol. Exp. Neurol..

[150] Liang X., Wu L., Hand T., Andreasson K. (2005). Prostaglandin D2 Mediates Neuronal Protection via the DP1 Receptor. J. Neurochem..

[151] Mohan S., Ahmad A.S., Glushakov A.V., Chambers C., Doré S. (2012). Putative Role of Prostaglandin Receptor in Intracerebral Hemorrhage. Front. Neurol..

[152] Biringer R.G. (2019). The Role of Eicosanoids in Alzheimer’s Disease. Int. J. Environ. Res. Public Health.

[153] Ahmad A.S., Ahmad M., Maruyama T., Narumiya S., Doré S. (2010). Prostaglandin D2 DP1 Receptor Is Beneficial in Ischemic Stroke and in Acute Exicitotoxicity in Young and Old Mice. Age.

[154] Bate C., Kempster S., Williams A. (2006). Prostaglandin D2 Mediates Neuronal Damage by Amyloid-Beta or Prions Which Activates Microglial Cells. Neuropharmacology.

[155] Ghosh A., Comerota M.M., Wan D., Chen F., Propson N.E., Hwang S.H., Hammock B.D., Zheng H. (2020). An Epoxide Hydrolase Inhibitor Reduces Neuroinflammation in a Mouse Model of Alzheimer’s Disease. Sci. Transl. Med..

[156] Wu Q., Lin M., Wu P., Zhao C., Yang S., Yu H., Xian W., Song J. (2022). TPPU Downregulates Oxidative Stress Damage and Induces BDNF Expression in PC-12 Cells. Comput. Math. Methods Med..

[157] Chen W., Wang M., Zhu M., Xiong W., Qin X., Zhu X. (2020). 14,15-Epoxyeicosatrienoic Acid Alleviates Pathology in a Mouse Model of Alzheimer’s Disease. J. Neurosci..

[158] Shi Z.-M., Han Y.-W., Han X.-H., Zhang K., Chang Y.-N., Hu Z.-M., Qi H.-X., Ting C., Zhen Z., Hong W. (2016). Upstream Regulators and Downstream Effectors of NF-ΚB in Alzheimer’s Disease. J. Neurol. Sci..

[159] Yang W., Liu Y., Xu Q.-Q., Xian Y.-F., Lin Z.-X. (2020). Sulforaphene Ameliorates Neuroinflammation and Hyperphosphorylated Tau Protein via Regulating the PI3K/Akt/GSK-3β Pathway in Experimental Models of Alzheimer’s Disease. Oxid. Med. Cell Longev..

[160] Wardyn J.D., Ponsford A.H., Sanderson C.M. (2015). Dissecting Molecular Cross-Talk between Nrf2 and NF-ΚB Response Pathways. Biochem. Soc. Trans..

[161] Innamorato N.G., Rojo A.I., García-Yagüe A.J., Yamamoto M., de Ceballos M.L., Cuadrado A. (2008). The Transcription Factor Nrf2 Is a Therapeutic Target against Brain Inflammation. J. Immunol..

[162] Danilov C.A., Chandrasekaran K., Racz J., Soane L., Zielke C., Fiskum G. (2009). Sulforaphane Protects Astrocytes against Oxidative Stress and Delayed Death Caused by Oxygen and Glucose Deprivation. Glia.

[163] Zhao F., Zhang J., Chang N. (2018). Epigenetic Modification of Nrf2 by Sulforaphane Increases the Antioxidative and Anti-Inflammatory Capacity in a Cellular Model of Alzheimer’s Disease. Eur. J. Pharmacol..

[164] Kim J. (2021). Pre-Clinical Neuroprotective Evidences and Plausible Mechanisms of Sulforaphane in Alzheimer’s Disease. Int. J. Mol. Sci..

[165] Kraft A.D., Johnson D.A., Johnson J.A. (2004). Nuclear Factor E2-Related Factor 2-Dependent Antioxidant Response Element Activation by Tert-Butylhydroquinone and Sulforaphane Occurring Preferentially in Astrocytes Conditions Neurons against Oxidative Insult. J. Neurosci..

[166] Vargas M.R., Johnson D.A., Sirkis D.W., Messing A., Johnson J.A. (2008). Nrf2 Activation in Astrocytes Protects against Neurodegeneration in Mouse Models of Familial Amyotrophic Lateral Sclerosis. J. Neurosci..

[167] Shi J.-Z., Zheng X.-M., Zhou Y.-F., Yun L.-Y., Luo D.-M., Hao J.-J., Liu P.-F., Zhang W.-K., Xu J.-K., Yan Y. (2022). Cornuside Is a Potential Agent against Alzheimer’s Disease via Orchestration of Reactive Astrocytes. Nutrients.

[168] Cabezas R., Avila-Rodriguez M., Vega-Vela N.E., Echeverria V., González J., Hidalgo O.A., Santos A.B., Aliev G., Barreto G.E. (2016). Growth Factors and Astrocytes Metabolism: Possible Roles for Platelet Derived Growth Factor. Med. Chem..

[169] Sycheva M., Sustarich J., Zhang Y., Selvaraju V., Geetha T., Gearing M., Babu J.R. (2019). Pro-Nerve Growth Factor Induces Activation of RhoA Kinase and Neuronal Cell Death. Brain Sci..

[170] Selles M.C., Fortuna J.T.S., Zappa-Villar M.F., de Faria Y.P.R., Souza A.S., Suemoto C.K., Leite R.E.P., Rodriguez R.D., Grinberg L.T., Reggiani P.C. (2020). Adenovirus-Mediated Transduction of Insulin-Like Growth Factor 1 Protects Hippocampal Neurons from the Toxicity of Aβ Oligomers and Prevents Memory Loss in an Alzheimer Mouse Model. Mol. Neurobiol..

[171] Albus E., Sinningen K., Winzer M., Thiele S., Baschant U., Hannemann A., Fantana J., Tausche A.-K., Wallaschofski H., Nauck M. (2016). Milk Fat Globule-Epidermal Growth Factor 8 (MFG-E8) Is a Novel Anti-Inflammatory Factor in Rheumatoid Arthritis in Mice and Humans. J. Bone Miner. Res..

[172] Kranich J., Krautler N.J., Falsig J., Ballmer B., Li S., Hutter G., Schwarz P., Moos R., Julius C., Miele G. (2010). Engulfment of Cerebral Apoptotic Bodies Controls the Course of Prion Disease in a Mouse Strain-Dependent Manner. J. Exp. Med..

[173] Xu X., Zhang A., Zhu Y., He W., Di W., Fang Y., Shi X. (2018). MFG-E8 Reverses Microglial-Induced Neurotoxic Astrocyte (A1) via NF-ΚB and PI3K-Akt Pathways. J. Cell Physiol..

[174] Kawabe K., Takano K., Moriyama M., Nakamura Y. (2018). Microglia Endocytose Amyloid β Through the Binding of Transglutaminase 2 and Milk Fat Globule EGF Factor 8 Protein. Neurochem. Res..

[175] Tamagno E., Guglielmotto M., Vasciaveo V., Tabaton M. (2021). Oxidative Stress and Beta Amyloid in Alzheimer’s Disease. Which Comes First: The Chicken or the Egg?. Antioxidants.

[176] Poljsak B. (2011). Strategies for Reducing or Preventing the Generation of Oxidative Stress. Oxid. Med. Cell Longev..

[177] Beydoun M.A., Beydoun H.A., Fanelli-Kuczmarski M.T., Weiss J., Hossain S., Canas J.A., Evans M.K., Zonderman A.B. (2022). Association of Serum Antioxidant Vitamins and Carotenoids with Incident Alzheimer Disease and All-Cause Dementia Among US Adults. Neurology.

[178] Ahmadinejad F., Geir Møller S., Hashemzadeh-Chaleshtori M., Bidkhori G., Jami M.-S. (2017). Molecular Mechanisms behind Free Radical Scavengers Function against Oxidative Stress. Antioxidants.

[179] Dajas F., Andrés A.-C.J., Florencia A., Carolina E., Felicia R.-M. (2013). Neuroprotective Actions of Flavones and Flavonols: Mechanisms and Relationship to Flavonoid Structural Features. Cent. Nerv. Syst. Agents Med. Chem..

[180] Nakajima A., Ohizumi Y. (2019). Potential Benefits of Nobiletin, A Citrus Flavonoid, against Alzheimer’s Disease and Parkinson’s Disease. Int. J. Mol. Sci..

[181] Yin N., Yao X., Zhou Q., Faiola F., Jiang G. (2015). Vitamin E Attenuates Silver Nanoparticle-Induced Effects on Body Weight and Neurotoxicity in Rats. Biochem. Biophys. Res. Commun..

[182] Behl C. (1999). Vitamin E and Other Antioxidants in Neuroprotection. Int. J. Vitam. Nutr. Res..

[183] Abedi Z., Khaza’ai H., Vidyadaran S., Mutalib M.S.A. (2017). The Modulation of NMDA and AMPA/Kainate Receptors by Tocotrienol-Rich Fraction and A-Tocopherol in Glutamate-Induced Injury of Primary Astrocytes. Biomedicines.

[184] Dysken M.W., Sano M., Asthana S., Vertrees J.E., Pallaki M., Llorente M., Love S., Schellenberg G.D., McCarten J.R., Malphurs J. (2014). Effect of Vitamin E and Memantine on Functional Decline in Alzheimer Disease: The TEAM-AD VA Cooperative Randomized Trial. JAMA.

[185] Kryscio R.J., Abner E.L., Caban-Holt A., Lovell M., Goodman P., Darke A.K., Yee M., Crowley J., Schmitt F.A. (2017). Association of Antioxidant Supplement Use and Dementia in the Prevention of Alzheimer’s Disease by Vitamin E and Selenium Trial (PREADViSE). JAMA Neurol..

[186] Farina N., Llewellyn D., Isaac M.G.E.K.N., Tabet N. (2017). Vitamin E for Alzheimer’s Dementia and Mild Cognitive Impairment. Cochrane Database Syst. Rev..

[187] Forman H.J., Zhang H. (2021). Targeting Oxidative Stress in Disease: Promise and Limitations of Antioxidant Therapy. Nat. Rev. Drug Discov..

[188] Persson T., Popescu B.O., Cedazo-Minguez A. (2014). Oxidative Stress in Alzheimer’s Disease: Why Did Antioxidant Therapy Fail?. Oxid. Med. Cell Longev..

[189] Yang E.-J., Kim H., Kim H.-S., Chang M.-J. (2021). Phloroglucinol Attenuates Oligomeric Amyloid Beta Peptide1-42-Induced Astrocytic Activation by Reducing Oxidative Stress. J. Pharmacol. Sci..

[190] Wang D., Gao F., Hu F., Wu J. (2022). Nobiletin Alleviates Astrocyte Activation and Oxidative Stress Induced by Hypoxia In Vitro. Molecules.

[191] Quincozes-Santos A., Bobermin L.D., Latini A., Wajner M., Souza D.O., Gonçalves C.-A., Gottfried C. (2013). Resveratrol Protects C6 Astrocyte Cell Line against Hydrogen Peroxide-Induced Oxidative Stress through Heme Oxygenase 1. PLoS ONE.

[192] Yu H., Yamashita T., Hu X., Bian Z., Hu X., Feng T., Tadokoro K., Morihara R., Abe K. (2022). Protective and anti-oxidative effects of curcumin and resveratrol on Aβ-oligomer-induced damage in the SH-SY5Y cell line. J. Neurol. Sci..

[193] Daverey A., Agrawal S.K. (2016). Curcumin Alleviates Oxidative Stress and Mitochondrial Dysfunction in Astrocytes. Neuroscience.

[194] López S., Martá M., Sequeda L.G., Celis C., Sutachan J.J., Albarracín S.L. (2017). Cytoprotective Action against Oxidative Stress in Astrocytes and Neurons by Bactris Guineensis (L.) H.E. Moore (Corozo) Fruit Extracts. Food Chem. Toxicol..

[195] Prah J., Winters A., Chaudhari K., Hersh J., Liu R., Yang S.-H. (2019). Cholesterol Sulfate Alters Astrocyte Metabolism and Provides Protection against Oxidative Stress. Brain Res..

[196] Lu C.-Y., Day C.H., Kuo C.-H., Wang T.-F., Ho T.-J., Lai P.-F., Chen R.-J., Yao C.-H., Viswanadha V.P., Kuo W.-W. (2022). Calycosin Alleviates H_2_O_2_-Induced Astrocyte Injury by Restricting Oxidative Stress through the Akt/Nrf2/HO-1 Signaling Pathway. Environ. Toxicol..

[197] Jeřábek J., Uliassi E., Guidotti L., Korábečný J., Soukup O., Sepsova V., Hrabinova M., Kuča K., Bartolini M., Peña-Altamira L.E. (2017). Tacrine-Resveratrol Fused Hybrids as Multi-Target-Directed Ligands against Alzheimer’s Disease. Eur. J. Med. Chem..

[198] Sun J., Xu S., Li H., Li L., Xu Z.-Q.D. (2019). Galanin Protects Rat Cortical Astrocyte from Oxidative Stress: Involvement of GalR2 and PERK1/2 Signal Pathway. Mediat. Inflamm..

[199] Bordet R., Gelé P., Duriez P., Fruchart J.-C. (2006). PPARs: A New Target for Neuroprotection. J. Neurol. Neurosurg. Psychiatry.

[200] Giampietro L., Gallorini M., De Filippis B., Amoroso R., Cataldi A., di Giacomo V. (2019). PPAR-γ Agonist GL516 Reduces Oxidative Stress and Apoptosis Occurrence in a Rat Astrocyte Cell Line. Neurochem. Int..

[201] Fracassi A., Marcatti M., Zolochevska O., Tabor N., Woltjer R., Moreno S., Taglialatela G. (2021). Oxidative Damage and Antioxidant Response in Frontal Cortex of Demented and Nondemented Individuals with Alzheimer’s Neuropathology. J. Neurosci..

[202] Bodega G., Alique M., Puebla L., Carracedo J., Ramírez R.M. (2019). Microvesicles: ROS Scavengers and ROS Producers. J. Extracell. Vesicles.

[203] Feng T., Yamashita T., Sasaki R., Tadokoro K., Matsumoto N., Hishikawa N., Abe K. (2021). Protective Effects of Edaravone on White Matter Pathology in a Novel Mouse Model of Alzheimer’s Disease with Chronic Cerebral Hypoperfusion. J. Cereb. Blood Flow Metab..

[204] Jiao S.-S., Yao X.-Q., Liu Y.-H., Wang Q.-H., Zeng F., Lu J.-J., Liu J., Zhu C., Shen L.-L., Liu C.-H. (2015). Edaravone Alleviates Alzheimer’s Disease-Type Pathologies and Cognitive Deficits. Proc. Natl. Acad. Sci. USA.

[205] Ren H., Ma L., Gong X., Xu C., Zhang Y., Ma M., Watanabe K., Wen J. (2019). Edaravone Exerts Brain Protective Function by Reducing the Expression of AQP4, APP and Aβ Proteins. Open Life Sci..

[206] Shanker G., Syversen T., Aschner J.L., Aschner M. (2005). Modulatory Effect of Glutathione Status and Antioxidants on Methylmercury-Induced Free Radical Formation in Primary Cultures of Cerebral Astrocytes. Brain Res. Mol. Brain Res..

[207] Matos M., Augusto E., Machado N.J., dos Santos-Rodrigues A., Cunha R.A., Agostinho P. (2012). Astrocytic Adenosine A2A Receptors Control the Amyloid-β Peptide-Induced Decrease of Glutamate Uptake. J. Alzheimer’s Dis..

[208] Fang T., Al Khleifat A., Meurgey J.-H., Jones A., Leigh P.N., Bensimon G., Al-Chalabi A. (2018). Stage at Which Riluzole Treatment Prolongs Survival in Patients with Amyotrophic Lateral Sclerosis: A Retrospective Analysis of Data from a Dose-Ranging Study. Lancet Neurol..

[209] Wang S.-J., Wang K.-Y., Wang W.-C. (2004). Mechanisms Underlying the Riluzole Inhibition of Glutamate Release from Rat Cerebral Cortex Nerve Terminals (Synaptosomes). Neuroscience.

[210] Carbone M., Duty S., Rattray M. (2012). Riluzole Elevates GLT-1 Activity and Levels in Striatal Astrocytes. Neurochem. Int..

[211] Lesuis S.L., Kaplick P.M., Lucassen P.J., Krugers H.J. (2019). Treatment with the Glutamate Modulator Riluzole Prevents Early Life Stress-Induced Cognitive Deficits and Impairments in Synaptic Plasticity in APPswe/PS1dE9 Mice. Neuropharmacology.

[212] Rothstein J.D., Patel S., Regan M.R., Haenggeli C., Huang Y.H., Bergles D.E., Jin L., Dykes Hoberg M., Vidensky S., Chung D.S. (2005). Beta-Lactam Antibiotics Offer Neuroprotection by Increasing Glutamate Transporter Expression. Nature.

[213] Zumkehr J., Rodriguez-Ortiz C.J., Cheng D., Kieu Z., Wai T., Hawkins C., Kilian J., Lim S.L., Medeiros R., Kitazawa M. (2015). Ceftriaxone Ameliorates Tau Pathology and Cognitive Decline via Restoration of Glial Glutamate Transporter in a Mouse Model of Alzheimer’s Disease. Neurobiol. Aging.

[214] Chotibut T., Davis R.W., Arnold J.C., Frenchek Z., Gurwara S., Bondada V., Geddes J.W., Salvatore M.F. (2014). Ceftriaxone Increases Glutamate Uptake and Reduces Striatal Tyrosine Hydroxylase Loss in 6-OHDA Parkinson’s Model. Mol. Neurobiol..

[215] Ho S.-C., Hsu C.-C., Pawlak C.R., Tikhonova M.A., Lai T.-J., Amstislavskaya T.G., Ho Y.-J. (2014). Effects of Ceftriaxone on the Behavioral and Neuronal Changes in an MPTP-Induced Parkinson’s Disease Rat Model. Behav. Brain Res..

[216] Bisht R., Kaur B., Gupta H., Prakash A. (2014). Ceftriaxone Mediated Rescue of Nigral Oxidative Damage and Motor Deficits in MPTP Model of Parkinson’s Disease in Rats. Neurotoxicology.

[217] Miller B.R., Dorner J.L., Shou M., Sari Y., Barton S.J., Sengelaub D.R., Kennedy R.T., Rebec G.V. (2008). Up-Regulation of GLT1 Expression Increases Glutamate Uptake and Attenuates the Huntington’s Disease Phenotype in the R6/2 Mouse. Neuroscience.

[218] Fan S., Li L., Xian X., Liu L., Gao J., Li W. (2021). Ceftriaxone Regulates Glutamate Production and Vesicular Assembly in Presynaptic Terminals through GLT-1 in APP/PS1 Mice. Neurobiol. Learn. Mem..

[219] Fan S., Xian X., Li L., Yao X., Hu Y., Zhang M., Li W. (2018). Ceftriaxone Improves Cognitive Function and Upregulates GLT-1-Related Glutamate-Glutamine Cycle in APP/PS1 Mice. J. Alzheimer’s Dis..

[220] Dzamba D., Honsa P., Anderova M. (2013). NMDA Receptors in Glial Cells: Pending Questions. Curr. Neuropharmacol..

[221] Mota S.I., Ferreira I.L., Rego A.C. (2014). Dysfunctional Synapse in Alzheimer’s Disease—A Focus on NMDA Receptors. Neuropharmacology.

[222] Lee M.-C., Ting K.K., Adams S., Brew B.J., Chung R., Guillemin G.J. (2010). Characterisation of the Expression of NMDA Receptors in Human Astrocytes. PLoS ONE.

[223] Li Y., Chang L., Song Y., Gao X., Roselli F., Liu J., Zhou W., Fang Y., Ling W., Li H. (2016). Astrocytic GluN2A and GluN2B Oppose the Synaptotoxic Effects of Amyloid-Β1-40 in Hippocampal Cells. J. Alzheimer’s Dis..

[224] Talantova M., Sanz-Blasco S., Zhang X., Xia P., Akhtar M.W., Okamoto S., Dziewczapolski G., Nakamura T., Cao G., Pratt A.E. (2013). Aβ Induces Astrocytic Glutamate Release, Extrasynaptic NMDA Receptor Activation, and Synaptic Loss. Proc. Natl. Acad. Sci. USA.

[225] Rush T., Buisson A. (2014). Reciprocal Disruption of Neuronal Signaling and Aβ Production Mediated by Extrasynaptic NMDA Receptors: A Downward Spiral. Cell Tissue Res..

[226] Palygin O., Lalo U., Verkhratsky A., Pankratov Y. (2010). Ionotropic NMDA and P2X1/5 Receptors Mediate Synaptically Induced Ca^2+^ Signalling in Cortical Astrocytes. Cell Calcium.

[227] Ueda Y., Doi T., Nagatomo K., Tokumaru J., Takaki M., Willmore L.J. (2007). Effect of Levetiracetam on Molecular Regulation of Hippocampal Glutamate and GABA Transporters in Rats with Chronic Seizures Induced by Amygdalar FeCl3 Injection. Brain Res..

[228] Sanz-Blasco S., Piña-Crespo J.C., Zhang X., McKercher S.R., Lipton S.A. (2016). Levetiracetam Inhibits Oligomeric Aβ-Induced Glutamate Release from Human Astrocytes. Neuroreport.

[229] Vossel K.A., Beagle A.J., Rabinovici G.D., Shu H., Lee S.E., Naasan G., Hegde M., Cornes S.B., Henry M.L., Nelson A.B. (2013). Seizures and Epileptiform Activity in the Early Stages of Alzheimer Disease. JAMA Neurol..

[230] Cumbo E., Ligori L.D. (2010). Levetiracetam, Lamotrigine, and Phenobarbital in Patients with Epileptic Seizures and Alzheimer’s Disease. Epilepsy Behav..

[231] Kovacic P., Somanathan R. (2010). Clinical Physiology and Mechanism of Dizocilpine (MK-801): Electron Transfer, Radicals, Redox Metabolites and Bioactivity. Oxid. Med. Cell Longev..

[232] Liu J., Chang L., Song Y., Li H., Wu Y. (2019). The Role of NMDA Receptors in Alzheimer’s Disease. Front. Neurosci..

[233] Abd El-Fatah I.M., Abdelrazek H.M.A., Ibrahim S.M., Abdallah D.M., El-Abhar H.S. (2021). Dimethyl Fumarate Abridged Tauo-/Amyloidopathy in a D-Galactose/Ovariectomy-Induced Alzheimer’s-like Disease: Modulation of AMPK/SIRT-1, AKT/CREB/BDNF, AKT/GSK-3β, Adiponectin/Adipo1R, and NF-ΚB/IL-1β/ROS Trajectories. Neurochem. Int..

[234] Pao P.-C., Tsai L.-H. (2021). Three Decades of Cdk5. J. Biomed. Sci..

[235] Shupp A., Casimiro M.C., Pestell R.G. (2017). Biological Functions of CDK5 and Potential CDK5 Targeted Clinical Treatments. Oncotarget.

[236] Posada-Duque R.A., Palacio-Castañeda V., Cardona-Gómez G.P. (2015). CDK5 Knockdown in Astrocytes Provide Neuroprotection as a Trophic Source via Rac1. Mol. Cell. Neurosci..

[237] Schaffer S., Kim H.W. (2018). Effects and Mechanisms of Taurine as a Therapeutic Agent. Biomol. Ther..

[238] Suárez L.M., Muñoz M.-D., Martín Del Río R., Solís J.M. (2016). Taurine Content in Different Brain Structures during Ageing: Effect on Hippocampal Synaptic Plasticity. Amino Acids.

[239] Ripps H., Shen W. (2012). Review: Taurine: A “Very Essential” Amino Acid. Mol. Vis..

[240] Vitvitsky V., Garg S.K., Banerjee R. (2011). Taurine Biosynthesis by Neurons and Astrocytes. J. Biol. Chem..

[241] Ochoa-de la Paz L., Zenteno E., Gulias-Cañizo R., Quiroz-Mercado H. (2019). Taurine and GABA Neurotransmitter Receptors, a Relationship with Therapeutic Potential?. Expert Rev. Neurother..

[242] Albrecht J., Schousboe A. (2005). Taurine Interaction with Neurotransmitter Receptors in the CNS: An Update. Neurochem. Res..

[243] Foos T.M., Wu J.-Y. (2002). The Role of Taurine in the Central Nervous System and the Modulation of Intracellular Calcium Homeostasis. Neurochem. Res..

[244] Ramírez-Guerrero S., Guardo-Maya S., Medina-Rincón G.J., Orrego-González E.E., Cabezas-Pérez R., González-Reyes R.E. (2022). Taurine and Astrocytes: A Homeostatic and Neuroprotective Relationship. Front. Mol. Neurosci..

[245] Louzada P.R., Paula Lima A.C., Mendonca-Silva D.L., Noël F., De Mello F.G., Ferreira S.T. (2004). Taurine Prevents the Neurotoxicity of Beta-Amyloid and Glutamate Receptor Agonists: Activation of GABA Receptors and Possible Implications for Alzheimer’s Disease and Other Neurological Disorders. FASEB J..

[246] Jang H., Lee S., Choi S.L., Kim H.Y., Baek S., Kim Y. (2017). Taurine Directly Binds to Oligomeric Amyloid-β and Recovers Cognitive Deficits in Alzheimer Model Mice. Adv. Exp. Med. Biol..

[247] Jahanshahi M., Nikmahzar E., Gorgani S. (2021). Taurine Can Decrease Phosphorylated Tau Protein Levels in Alzheimer’s Model Rats’ Brains. Kathmandu Univ. Med. J..

[248] Reeta K.H., Singh D., Gupta Y.K. (2017). Chronic Treatment with Taurine after Intracerebroventricular Streptozotocin Injection Improves Cognitive Dysfunction in Rats by Modulating Oxidative Stress, Cholinergic Functions and Neuroinflammation. Neurochem. Int..

[249] Kim H.Y., Kim H.V., Yoon J.H., Kang B.R., Cho S.M., Lee S., Kim J.Y., Kim J.W., Cho Y., Woo J. (2014). Taurine in Drinking Water Recovers Learning and Memory in the Adult APP/PS1 Mouse Model of Alzheimer’s Disease. Sci. Rep..

[250] Rafiee Z., García-Serrano A.M., Duarte J.M. (2022). Taurine Supplementation as a Neuroprotective Strategy upon Brain Dysfunction in Metabolic Syndrome and Diabetes. Nutrients.

[251] Liu Q., Zhang J. (2014). Lipid Metabolism in Alzheimer’s Disease. Neurosci. Bull..

[252] Raha S., Ghosh A., Dutta D., Patel D.R., Pahan K. (2021). Activation of PPARα Enhances Astroglial Uptake and Degradation of β-Amyloid. Sci. Signal..

[253] Jeong W., Lee H., Cho S., Seo J. (2019). ApoE4-Induced Cholesterol Dysregulation and Its Brain Cell Type-Specific Implications in the Pathogenesis of Alzheimer’s Disease. Mol. Cells.

[254] Konings S.C., Torres-Garcia L., Martinsson I., Gouras G.K. (2021). Astrocytic and Neuronal Apolipoprotein E Isoforms Differentially Affect Neuronal Excitability. Front. Neurosci..

[255] Colton C.A., Brown C.M., Cook D., Needham L.K., Xu Q., Czapiga M., Saunders A.M., Schmechel D.E., Rasheed K., Vitek M.P. (2002). APOE and the Regulation of Microglial Nitric Oxide Production: A Link between Genetic Risk and Oxidative Stress. Neurobiol. Aging.

[256] Safieh M., Korczyn A.D., Michaelson D.M. (2019). ApoE4: An Emerging Therapeutic Target for Alzheimer’s Disease. BMC Med..

[257] Arboleda-Velasquez J.F., Lopera F., O’Hare M., Delgado-Tirado S., Marino C., Chmielewska N., Saez-Torres K.L., Amarnani D., Schultz A.P., Sperling R.A. (2019). Resistance to Autosomal Dominant Alzheimer’s Disease in an APOE3 Christchurch Homozygote: A Case Report. Nat. Med..

[258] Lin Y.-T., Seo J., Gao F., Feldman H.M., Wen H.-L., Penney J., Cam H.P., Gjoneska E., Raja W.K., Cheng J. (2018). APOE4 Causes Widespread Molecular and Cellular Alterations Associated with Alzheimer’s Disease Phenotypes in Human IPSC-Derived Brain Cell Types. Neuron.

[259] Mamun A.A., Uddin M., Bashar B., Fahim M., Zaman S., Begum Y., Bulbul I.J., Islam M., Sarwar M., Mathew B. (2020). Molecular Insight into the Therapeutic Promise of Targeting APOE4 for Alzheimer’s Disease. Oxid. Med. Cell. Longev..

[260] Xiong M., Jiang H., Serrano J.R., Gonzales E.R., Wang C., Gratuze M., Hoyle R., Bien-Ly N., Silverman A.P., Sullivan P.M. (2021). APOE Immunotherapy Reduces Cerebral Amyloid Angiopathy and Amyloid Plaques While Improving Cerebrovascular Function. Sci. Transl. Med..

[261] Sadowski M., Pankiewicz J., Scholtzova H., Ripellino J.A., Li Y., Schmidt S.D., Mathews P.M., Fryer J.D., Holtzman D.M., Sigurdsson E.M. (2004). A Synthetic Peptide Blocking the Apolipoprotein E/β-Amyloid Binding Mitigates β-Amyloid Toxicity and Fibril Formation in Vitro and Reduces β-Amyloid Plaques in Transgenic Mice. Am. J. Pathol..

[262] Liu S., Breitbart A., Sun Y., Mehta P.D., Boutajangout A., Scholtzova H., Wisniewski T. (2014). Blocking the Apolipoprotein E/Amyloid β Interaction in Triple Transgenic Mice Ameliorates Alzheimer’s Disease Related Amyloid β and Tau Pathology. J. Neurochem..

[263] Terwel D., Steffensen K.R., Verghese P.B., Kummer M.P., Gustafsson J.-Å., Holtzman D.M., Heneka M.T. (2011). Critical Role of Astroglial Apolipoprotein E and Liver X Receptor-α Expression for Microglial Aβ Phagocytosis. J. Neurosci..

[264] Skerrett R., Pellegrino M.P., Casali B.T., Taraboanta L., Landreth G.E. (2015). Combined Liver X Receptor/Peroxisome Proliferator-Activated Receptor γ Agonist Treatment Reduces Amyloid β Levels and Improves Behavior in Amyloid Precursor Protein/Presenilin 1 Mice. J. Biol. Chem..

[265] Muñoz-Cabrera J.M., Sandoval-Hernández A.G., Niño A., Báez T., Bustos-Rangel A., Cardona-Gómez G.P., Múnera A., Arboleda G. (2019). Bexarotene Therapy Ameliorates Behavioral Deficits and Induces Functional and Molecular Changes in Very-Old Triple Transgenic Mice Model of Alzheimer´s Disease. PLoS ONE.

[266] Takeuchi M., Yamagishi S. (2008). Possible Involvement of Advanced Glycation End-Products (AGEs) in the Pathogenesis of Alzheimer’s Disease. Curr. Pharm. Des..

[267] Rungratanawanich W., Qu Y., Wang X., Essa M.M., Song B.-J. (2021). Advanced Glycation End Products (AGEs) and Other Adducts in Aging-Related Diseases and Alcohol-Mediated Tissue Injury. Exp. Mol. Med..

[268] González-Reyes R.E., Aliev G., Ávila-Rodrigues M., Barreto G.E. (2016). Alterations in Glucose Metabolism on Cognition: A Possible Link Between Diabetes and Dementia. Curr. Pharm. Des..

[269] Choi B.-R., Cho W.-H., Kim J., Lee H.J., Chung C., Jeon W.K., Han J.-S. (2014). Increased Expression of the Receptor for Advanced Glycation End Products in Neurons and Astrocytes in a Triple Transgenic Mouse Model of Alzheimer’s Disease. Exp. Mol. Med..

[270] Srikanth V., Maczurek A., Phan T., Steele M., Westcott B., Juskiw D., Münch G. (2011). Advanced Glycation Endproducts and Their Receptor RAGE in Alzheimer’s Disease. Neurobiol. Aging.

[271] Kamynina A., Esteras N., Koroev D.O., Angelova P.R., Volpina O.M., Abramov A.Y. (2021). Activation of RAGE Leads to the Release of Glutamate from Astrocytes and Stimulates Calcium Signal in Neurons. J. Cell. Physiol..

[272] Zhang C., Wang L., Xu Y., Huang Y., Huang J., Zhu J., Wang W., Li W., Sun A., Li X. (2022). Discovery of Novel Dual RAGE/SERT Inhibitors for the Potential Treatment of the Comorbidity of Alzheimer’s Disease and Depression. Eur. J. Med. Chem..

[273] Xue J., Jia P., Zhang D., Yao Z. (2021). TTP488 Ameliorates NLRP3-Associated Inflammation, Viability, Apoptosis, and ROS Production in an Alzheimer’s Disease Cell Model by Mediating the JAK1/STAT3/NFκB/IRF3 Pathway. Cell Biochem. Funct..

[274] Yang L., Liu Y., Wang Y., Li J., Liu N. (2021). Azeliragon Ameliorates Alzheimer’s Disease via the Janus Tyrosine Kinase and Signal Transducer and Activator of Transcription Signaling Pathway. Clinics.

[275] Muoio V., Persson P.B., Sendeski M.M. (2014). The Neurovascular Unit—Concept Review. Acta Physiol..

[276] Abbott N.J., Rönnbäck L., Hansson E. (2006). Astrocyte-Endothelial Interactions at the Blood-Brain Barrier. Nat. Rev. Neurosci..

[277] Liu C.-Y., Yang Y., Ju W.-N., Wang X., Zhang H.-L. (2018). Emerging Roles of Astrocytes in Neuro-Vascular Unit and the Tripartite Synapse with Emphasis on Reactive Gliosis in the Context of Alzheimer’s Disease. Front. Cell Neurosci..

[278] Sweeney M.D., Kisler K., Montagne A., Toga A.W., Zlokovic B.V. (2018). The Role of Brain Vasculature in Neurodegenerative Disorders. Nat. Neurosci..

[279] Sweeney M.D., Sagare A.P., Zlokovic B.V. (2018). Blood-Brain Barrier Breakdown in Alzheimer Disease and Other Neurodegenerative Disorders. Nat. Rev. Neurol..

[280] Yu X., Ji C., Shao A. (2020). Neurovascular Unit Dysfunction and Neurodegenerative Disorders. Front. Neurosci..

[281] Zapata-Acevedo J.F., García-Pérez V., Cabezas-Pérez R., Losada-Barragán M., Vargas-Sánchez K., González-Reyes R.E. (2022). Laminin as a Biomarker of Blood-Brain Barrier Disruption under Neuroinflammation: A Systematic Review. Int. J. Mol. Sci..

[282] Sweeney M.D., Montagne A., Sagare A.P., Nation D.A., Schneider L.S., Chui H.C., Harrington M.G., Pa J., Law M., Wang D.J.J. (2019). Vascular Dysfunction—The Disregarded Partner of Alzheimer’s Disease. Alzheimer’s Dement..

[283] Kirabali T., Rust R., Rigotti S., Siccoli A., Nitsch R.M., Kulic L. (2020). Distinct Changes in All Major Components of the Neurovascular Unit across Different Neuropathological Stages of Alzheimer’s Disease. Brain Pathol..

[284] Yáñez-Mó M., Siljander P.R.-M., Andreu Z., Zavec A.B., Borràs F.E., Buzas E.I., Buzas K., Casal E., Cappello F., Carvalho J. (2015). Biological Properties of Extracellular Vesicles and Their Physiological Functions. J. Extracell. Vesicles.

[285] González-Molina L.A., Villar-Vesga J., Henao-Restrepo J., Villegas A., Lopera F., Cardona-Gómez G.P., Posada-Duque R. (2021). Extracellular Vesicles From 3xTg-AD Mouse and Alzheimer’s Disease Patient Astrocytes Impair Neuroglial and Vascular Components. Front. Aging Neurosci..

[286] Bell R.D., Winkler E.A., Singh I., Sagare A.P., Deane R., Wu Z., Holtzman D.M., Betsholtz C., Armulik A., Sallstrom J. (2012). Apolipoprotein E Controls Cerebrovascular Integrity via Cyclophilin, A. Nature.

[287] Natale G., Limanaqi F., Busceti C.L., Mastroiacovo F., Nicoletti F., Puglisi-Allegra S., Fornai F. (2021). Glymphatic System as a Gateway to Connect Neurodegeneration from Periphery to CNS. Front. Neurosci..

[288] Xie L., Kang H., Xu Q., Chen M.J., Liao Y., Thiyagarajan M., O’Donnell J., Christensen D.J., Nicholson C., Iliff J.J. (2013). Sleep Drives Metabolite Clearance from the Adult Brain. Science.

[289] Reddy O.C., van der Werf Y.D. (2020). The Sleeping Brain: Harnessing the Power of the Glymphatic System through Lifestyle Choices. Brain Sci..

[290] Hablitz L.M., Vinitsky H.S., Sun Q., Stæger F.F., Sigurdsson B., Mortensen K.N., Lilius T.O., Nedergaard M. (2019). Increased Glymphatic Influx Is Correlated with High EEG Delta Power and Low Heart Rate in Mice under Anesthesia. Sci. Adv..

[291] Jessen N.A., Munk A.S.F., Lundgaard I., Nedergaard M. (2015). The Glymphatic System: A Beginner’s Guide. Neurochem. Res..

[292] Shokri-Kojori E., Wang G.-J., Wiers C.E., Demiral S.B., Guo M., Kim S.W., Lindgren E., Ramirez V., Zehra A., Freeman C. (2018). β-Amyloid Accumulation in the Human Brain after One Night of Sleep Deprivation. Proc. Natl. Acad. Sci. USA.

[293] Simon M., Wang M.X., Ismail O., Braun M., Schindler A.G., Reemmer J., Wang Z., Haveliwala M.A., O’Boyle R.P., Han W.Y. (2022). Loss of Perivascular Aquaporin-4 Localization Impairs Glymphatic Exchange and Promotes Amyloid β Plaque Formation in Mice. Alzheimer’s Res. Ther..

[294] Feng W., Zhang Y., Wang Z., Xu H., Wu T., Marshall C., Gao J., Xiao M. (2020). Microglia Prevent Beta-Amyloid Plaque Formation in the Early Stage of an Alzheimer’s Disease Mouse Model with Suppression of Glymphatic Clearance. Alzheimer’s Res. Ther..

[295] Ren H., Luo C., Feng Y., Yao X., Shi Z., Liang F., Kang J.X., Wan J.-B., Pei Z., Su H. (2017). Omega-3 Polyunsaturated Fatty Acids Promote Amyloid-β Clearance from the Brain through Mediating the Function of the Glymphatic System. FASEB J..

[296] Zhang X., O’Callaghan P., Li H., Tan Y., Zhang G., Barash U., Wang X., Lannfelt L., Vlodavsky I., Lindahl U. (2021). Heparanase Overexpression Impedes Perivascular Clearance of Amyloid-β from Murine Brain: Relevance to Alzheimer’s Disease. Acta Neuropathol. Commun..

[297] Yang J., Zhang R., Shi C., Mao C., Yang Z., Suo Z., Torp R., Xu Y. (2017). AQP4 Association with Amyloid Deposition and Astrocyte Pathology in the Tg-ArcSwe Mouse Model of Alzheimer’s Disease. J. Alzheimer’s Dis..

[298] Zhang B., Li W., Zhuo Y., Xiang H., Li W., Liu H., Xie L., Gao Q., Tan S. (2021). L-3-n-Butylphthalide Effectively Improves the Glymphatic Clearance and Reduce Amyloid-β Deposition in Alzheimer’s Transgenic Mice. J. Mol. Neurosci..

[299] Wang D., Chen F., Han Z., Yin Z., Ge X., Lei P. (2021). Relationship Between Amyloid-β Deposition and Blood-Brain Barrier Dysfunction in Alzheimer’s Disease. Front. Cell Neurosci..

[300] Arélin K., Kinoshita A., Whelan C.M., Irizarry M.C., Rebeck G.W., Strickland D.K., Hyman B.T. (2002). LRP and Senile Plaques in Alzheimer’s Disease: Colocalization with Apolipoprotein E and with Activated Astrocytes. Brain Res. Mol. Brain Res..

[301] Seok H., Lee M., Shin E., Yun M.R., Lee Y., Moon J.H., Kim E., Lee P.H., Lee B.-W., Kang E.S. (2019). Low-Dose Pioglitazone Can Ameliorate Learning and Memory Impairment in a Mouse Model of Dementia by Increasing LRP1 Expression in the Hippocampus. Sci. Rep..

[302] Hellström-Lindahl E., Ravid R., Nordberg A. (2008). Age-Dependent Decline of Neprilysin in Alzheimer’s Disease and Normal Brain: Inverse Correlation with Aβ Levels. Neurobiol. Aging.

[303] Apelt J., Ach K., Schliebs R. (2003). Aging-Related down-Regulation of Neprilysin, a Putative β-Amyloid-Degrading Enzyme, in Transgenic Tg2576 Alzheimer-like Mouse Brain Is Accompanied by an Astroglial Upregulation in the Vicinity of β-Amyloid Plaques. Neurosci. Lett..

[304] Saito T., Iwata N., Tsubuki S., Takaki Y., Takano J., Huang S.-M., Suemoto T., Higuchi M., Saido T.C. (2005). Somatostatin Regulates Brain Amyloid Beta Peptide Abeta42 through Modulation of Proteolytic Degradation. Nat. Med..

[305] Yamamoto N., Shibata M., Ishikuro R., Tanida M., Taniguchi Y., Ikeda-Matsuo Y., Sobue K. (2017). Epigallocatechin Gallate Induces Extracellular Degradation of Amyloid β-Protein by Increasing Neprilysin Secretion from Astrocytes through Activation of ERK and PI3K Pathways. Neuroscience.

[306] Brezovakova V., Sykova E., Jadhav S. (2022). Astrocytes Derived from Familial and Sporadic Alzheimer’s Disease IPSCs Show Altered Calcium Signaling and Respond Differently to Misfolded Protein Tau. Cells.

[307] Li M.-Z., Zheng L.-J., Shen J., Li X.-Y., Zhang Q., Bai X., Wang Q.-S., Ji J.-G. (2018). SIRT1 Facilitates Amyloid Beta Peptide Degradation by Upregulating Lysosome Number in Primary Astrocytes. Neural Regen. Res..

[308] Lee Y.F., Lariviere L., Russ A.N., Choi S.-Z., Bacskai B.J., Kastanenka K.V. (2021). Novel Botanical Therapeutic NB-02 Effectively Treats Alzheimer’s Neuropathophysiology in an APP/PS1 Mouse Model. eNeuro.

[309] Pagnier G.J., Kastanenka K.V., Sohn M., Choi S., Choi S., Soh H., Bacskai B.J. (2018). Novel Botanical Drug DA-9803 Prevents Deficits in Alzheimer’s Mouse Models. Alzheimer’s Res. Ther..

[310] Guerra-Gomes S., Sousa N., Pinto L., Oliveira J.F. (2017). Functional Roles of Astrocyte Calcium Elevations: From Synapses to Behavior. Front. Cell Neurosci..

[311] Semyanov A., Henneberger C., Agarwal A. (2020). Making Sense of Astrocytic Calcium Signals—From Acquisition to Interpretation. Nat. Rev. Neurosci..

[312] Zorec R., Araque A., Carmignoto G., Haydon P.G., Verkhratsky A., Parpura V. (2012). Astroglial Excitability and Gliotransmission: An Appraisal of Ca^2+^ as a Signalling Route. ASN Neuro.

[313] Navarrete M., Perea G., Maglio L., Pastor J., García de Sola R., Araque A. (2013). Astrocyte Calcium Signal and Gliotransmission in Human Brain Tissue. Cereb. Cortex.

[314] Jackson J.G., Robinson M.B. (2018). Regulation of Mitochondrial Dynamics in Astrocytes: Mechanisms, Consequences, and Unknowns. Glia.

[315] Okubo Y. (2020). Astrocytic Ca^2+^ Signaling Mediated by the Endoplasmic Reticulum in Health and Disease. J. Pharmacol. Sci..

[316] Shigetomi E., Tong X., Kwan K.Y., Corey D.P., Khakh B.S. (2011). TRPA1 Channels Regulate Astrocyte Resting Calcium Levels and Inhibitory Synapse Efficacy via GAT-3. Nat. Neurosci..

[317] Toescu E.C., Verkhratsky A. (2007). The Importance of Being Subtle: Small Changes in Calcium Homeostasis Control Cognitive Decline in Normal Aging. Aging Cell.

[318] Cascella R., Cecchi C. (2021). Calcium Dyshomeostasis in Alzheimer’s Disease Pathogenesis. Int. J. Mol. Sci..

[319] Parpura V., Grubišić V., Verkhratsky A. (2011). Ca^2+^ Sources for the Exocytotic Release of Glutamate from Astrocytes. Biochim. Biophys. Acta.

[320] Chiarini A., Armato U., Liu D., Dal Prà I. (2016). Calcium-Sensing Receptors of Human Neural Cells Play Crucial Roles in Alzheimer’s Disease. Front. Physiol..

[321] Magi S., Castaldo P., Macrì M.L., Maiolino M., Matteucci A., Bastioli G., Gratteri S., Amoroso S., Lariccia V. (2016). Intracellular Calcium Dysregulation: Implications for Alzheimer’s Disease. BioMed Res. Int..

[322] Armato U., Chiarini A., Chakravarthy B., Chioffi F., Pacchiana R., Colarusso E., Whitfield J.F., Dal Prà I. (2013). Calcium-Sensing Receptor Antagonist (Calcilytic) NPS 2143 Specifically Blocks the Increased Secretion of Endogenous Aβ42 Prompted by Exogenous Fibrillary or Soluble Aβ25-35 in Human Cortical Astrocytes and Neurons-Therapeutic Relevance to Alzheimer’s Disease. Biochim. Biophys. Acta.

[323] Chiarini A., Armato U., Gardenal E., Gui L., Dal Prà I. (2017). Amyloid β-Exposed Human Astrocytes Overproduce Phospho-Tau and Overrelease It within Exosomes, Effects Suppressed by Calcilytic NPS 2143—Further Implications for Alzheimer’s Therapy. Front. Neurosci..

[324] Chiarini A., Armato U., Hu P., Dal Prà I. (2020). CaSR Antagonist (Calcilytic) NPS 2143 Hinders the Release of Neuroinflammatory IL-6, Soluble ICAM-1, RANTES, and MCP-2 from Aβ-Exposed Human Cortical Astrocytes. Cells.

[325] Liu L., Chen M., Lin K., Xiang X., Yang J., Zheng Y., Xiong X., Zhu S. (2020). TRPC6 Attenuates Cortical Astrocytic Apoptosis and Inflammation in Cerebral Ischemic/Reperfusion Injury. Front. Cell Dev. Biol..

[326] Lu R., He Q., Wang J. (2017). TRPC Channels and Alzheimer’s Disease. Adv. Exp. Med. Biol..

[327] Griffith T.N., Varela-Nallar L., Dinamarca M.C., Inestrosa N.C. (2010). Neurobiological Effects of Hyperforin and Its Potential in Alzheimer’s Disease Therapy. Curr. Med. Chem..

[328] Zhang H., Sun S., Wu L., Pchitskaya E., Zakharova O., Fon Tacer K., Bezprozvanny I. (2016). Store-Operated Calcium Channel Complex in Postsynaptic Spines: A New Therapeutic Target for Alzheimer’s Disease Treatment. J. Neurosci..

[329] Huang W., Cheng P., Yu K., Han Y., Song M., Li Y. (2017). Hyperforin Attenuates Aluminum-Induced Aβ Production and Tau Phosphorylation via Regulating Akt/GSK-3β Signaling Pathway in PC12 Cells. Biomed. Pharmacother..

[330] Cerpa W., Hancke J.L., Morazzoni P., Bombardelli E., Riva A., Marin P.P., Inestrosa N.C. (2010). The Hyperforin Derivative IDN5706 Occludes Spatial Memory Impairments and Neuropathological Changes in a Double Transgenic Alzheimer’s Mouse Model. Curr. Alzheimer Res..

[331] Inestrosa N.C., Tapia-Rojas C., Griffith T.N., Carvajal F.J., Benito M.J., Rivera-Dictter A., Alvarez A.R., Serrano F.G., Hancke J.L., Burgos P.V. (2011). Tetrahydrohyperforin Prevents Cognitive Deficit, Aβ Deposition, Tau Phosphorylation and Synaptotoxicity in the APPswe/PSEN1ΔE9 Model of Alzheimer’s Disease: A Possible Effect on APP Processing. Transl. Psychiatry.

[332] Bernal-Chico A., Tepavcevic V., Manterola A., Utrilla C., Matute C., Mato S. (2022). Endocannabinoid Signaling in Brain Diseases: Emerging Relevance of Glial Cells. Glia.

[333] Gutiérrez-Rodríguez A., Bonilla-Del Río I., Puente N., Gómez-Urquijo S.M., Fontaine C.J., Egaña-Huguet J., Elezgarai I., Ruehle S., Lutz B., Robin L.M. (2018). Localization of the Cannabinoid Type-1 Receptor in Subcellular Astrocyte Compartments of Mutant Mouse Hippocampus. Glia.

[334] Achicallende S., Bonilla-Del Río I., Serrano M., Mimenza A., Lekunberri L., Anaut-Lusar I., Puente N., Gerrikagoitia I., Grandes P. (2022). GLAST versus GFAP as Astroglial Marker for the Subcellular Study of Cannabinoid CB1 Receptors in Astrocytes. Histochem. Cell Biol..

[335] López A., Aparicio N., Pazos M.R., Grande M.T., Barreda-Manso M.A., Benito-Cuesta I., Vázquez C., Amores M., Ruiz-Pérez G., García-García E. (2018). Cannabinoid CB2 Receptors in the Mouse Brain: Relevance for Alzheimer’s Disease. J. Neuroinflamm..

[336] Aguirre-Rueda D., Guerra-Ojeda S., Aldasoro M., Iradi A., Obrador E., Mauricio M.D., Vila J.M., Marchio P., Valles S.L. (2015). WIN 55,212-2, Agonist of Cannabinoid Receptors, Prevents Amyloid Β1-42 Effects on Astrocytes in Primary Culture. PLoS ONE.

[337] Esposito G., Scuderi C., Savani C., Steardo L., De Filippis D., Cottone P., Iuvone T., Cuomo V., Steardo L. (2007). Cannabidiol in Vivo Blunts Beta-Amyloid Induced Neuroinflammation by Suppressing IL-1beta and INOS Expression. Br. J. Pharmacol..

[338] Wright D.C., Geiger P.C., Han D.-H., Jones T.E., Holloszy J.O. (2007). Calcium Induces Increases in Peroxisome Proliferator-Activated Receptor Gamma Coactivator-1alpha and Mitochondrial Biogenesis by a Pathway Leading to P38 Mitogen-Activated Protein Kinase Activation. J. Biol. Chem..

[339] Rivera A., Vanzulli I., Butt A.M. (2016). A Central Role for ATP Signalling in Glial Interactions in the CNS. Curr. Drug Targets.

[340] Erb L., Cao C., Ajit D., Weisman G.A. (2015). P2Y Receptors in Alzheimer’s Disease. Biol. Cell.

[341] Delekate A., Füchtemeier M., Schumacher T., Ulbrich C., Foddis M., Petzold G.C. (2014). Metabotropic P2Y1 Receptor Signalling Mediates Astrocytic Hyperactivity in Vivo in an Alzheimer’s Disease Mouse Model. Nat. Commun..

[342] Reichenbach N., Delekate A., Breithausen B., Keppler K., Poll S., Schulte T., Peter J., Plescher M., Hansen J.N., Blank N. (2018). P2Y1 Receptor Blockade Normalizes Network Dysfunction and Cognition in an Alzheimer’s Disease Model. J. Exp. Med..

[343] Zhou J.-N., Liu R.-Y., Kamphorst W., Hofman M.A., Swaab D.F. (2003). Early Neuropathological Alzheimer’s Changes in Aged Individuals Are Accompanied by Decreased Cerebrospinal Fluid Melatonin Levels. J. Pineal Res..

[344] Gonzalez A. (2021). Antioxidants and Neuron-Astrocyte Interplay in Brain Physiology: Melatonin, a Neighbor to Rely On. Neurochem. Res..

[345] Zhu L.Q., Wang S.H., Ling Z.Q., Wang D.L., Wang J.-Z. (2004). Effect of Inhibiting Melatonin Biosynthesis on Spatial Memory Retention and Tau Phosphorylation in Rat. J. Pineal Res..

[346] Zhang S., Wang P., Ren L., Hu C., Bi J. (2016). Protective Effect of Melatonin on Soluble Aβ1-42-Induced Memory Impairment, Astrogliosis, and Synaptic Dysfunction via the Musashi1/Notch1/Hes1 Signaling Pathway in the Rat Hippocampus. Alzheimer’s Res. Ther..

[347] Chung S.-Y., Han S.-H. (2003). Melatonin Attenuates Kainic Acid-Induced Hippocampal Neurodegeneration and Oxidative Stress through Microglial Inhibition. J. Pineal Res..

[348] Olivier P., Fontaine R.H., Loron G., Van Steenwinckel J., Biran V., Massonneau V., Kaindl A., Dalous J., Charriaut-Marlangue C., Aigrot M.-S. (2009). Melatonin Promotes Oligodendroglial Maturation of Injured White Matter in Neonatal Rats. PLoS ONE.

[349] Liu J., Clough S.J., Hutchinson A.J., Adamah-Biassi E.B., Popovska-Gorevski M., Dubocovich M.L. (2016). MT1 and MT2 Melatonin Receptors: A Therapeutic Perspective. Annu. Rev. Pharmacol. Toxicol..

[350] Srinivasan V., Kaur C., Pandi-Perumal S., Brown G.M., Cardinali D.P. (2010). Melatonin and Its Agonist Ramelteon in Alzheimer’s Disease: Possible Therapeutic Value. Int. J. Alzheimer’s Dis..

[351] Xiang J., Zhu W., Yang F., Yu Z.-H., Cai M., Li X.-T., Zhang J.-S., Zhang W., Cai D.-F. (2020). Melatonin-Induced ApoE Expression in Mouse Astrocytes Protects Endothelial Cells from OGD-R Induced Injuries. Transl. Psychiatry.

[352] Wang L.-Y., Pei J., Zhan Y.-J., Cai Y.-W. (2020). Overview of Meta-Analyses of Five Non-Pharmacological Interventions for Alzheimer’s Disease. Front. Aging Neurosci..

[353] Meng Q., Lin M.-S., Tzeng I.-S. (2020). Relationship Between Exercise and Alzheimer’s Disease: A Narrative Literature Review. Front. Neurosci..

[354] Livingston G., Huntley J., Sommerlad A., Ames D., Ballard C., Banerjee S., Brayne C., Burns A., Cohen-Mansfield J., Cooper C. (2020). Dementia Prevention, Intervention, and Care: 2020 Report of the Lancet Commission. Lancet.

[355] Jahangiri Z., Gholamnezhad Z., Hosseini M. (2019). Neuroprotective Effects of Exercise in Rodent Models of Memory Deficit and Alzheimer’s. Metab. Brain Dis..

[356] Belaya I., Ivanova M., Sorvari A., Ilicic M., Loppi S., Koivisto H., Varricchio A., Tikkanen H., Walker F.R., Atalay M. (2020). Astrocyte Remodeling in the Beneficial Effects of Long-Term Voluntary Exercise in Alzheimer’s Disease. J. Neuroinflamm..

[357] Koppel S.J., Pei D., Wilkins H.M., Weidling I.W., Wang X., Menta B.W., Perez-Ortiz J., Kalani A., Manley S., Novikova L. (2021). A Ketogenic Diet Differentially Affects Neuron and Astrocyte Transcription. J. Neurochem..

[358] Horner S., Berger L., Gibas K. (2020). Nutritional Ketosis and Photobiomodulation Remediate Mitochondria Warding off Alzheimer’s Disease in a Diabetic, ApoE4+ Patient with Mild Cognitive Impairment: A Case Report. Photodiagn. Photodyn. Ther..

[359] Phillips M.C.L., Deprez L.M., Mortimer G.M.N., Murtagh D.K.J., McCoy S., Mylchreest R., Gilbertson L.J., Clark K.M., Simpson P.V., McManus E.J. (2021). Randomized Crossover Trial of a Modified Ketogenic Diet in Alzheimer’s Disease. Alzheimer’s Res. Ther..

[360] Nardone R., Höller Y., Tezzon F., Christova M., Schwenker K., Golaszewski S., Trinka E., Brigo F. (2015). Neurostimulation in Alzheimer’s Disease: From Basic Research to Clinical Applications. Neurol. Sci..

[361] Lin Y., Jin J., Lv R., Luo Y., Dai W., Li W., Tang Y., Wang Y., Ye X., Lin W.-J. (2021). Repetitive Transcranial Magnetic Stimulation Increases the Brain’s Drainage Efficiency in a Mouse Model of Alzheimer’s Disease. Acta Neuropathol. Commun..

[362] Tsoy A., Saliev T., Abzhanova E., Turgambayeva A., Kaiyrlykyzy A., Akishev M., Saparbayev S., Umbayev B., Askarova S. (2019). The Effects of Mobile Phone Radiofrequency Electromagnetic Fields on β-Amyloid-Induced Oxidative Stress in Human and Rat Primary Astrocytes. Neuroscience.

[363] Koch G., Bonnì S., Pellicciari M.C., Casula E.P., Mancini M., Esposito R., Ponzo V., Picazio S., Di Lorenzo F., Serra L. (2018). Transcranial Magnetic Stimulation of the Precuneus Enhances Memory and Neural Activity in Prodromal Alzheimer’s Disease. Neuroimage.

[364] Ahmed M.A., Darwish E.S., Khedr E.M., El Serogy Y.M., Ali A.M. (2012). Effects of Low versus High Frequencies of Repetitive Transcranial Magnetic Stimulation on Cognitive Function and Cortical Excitability in Alzheimer’s Dementia. J. Neurol..

[365] Song Y.-Y., Xu W.-T., Zhang X.-C., Ni G.-X. (2020). Mechanisms of Electroacupuncture on Alzheimer’s Disease: A Review of Animal Studies. Chin. J. Integr. Med..

[366] Liang P.-Z., Li L., Zhang Y.-N., Shen Y., Zhang L.-L., Zhou J., Wang Z.-J., Wang S., Yang S. (2021). Electroacupuncture Improves Clearance of Amyloid-β through the Glymphatic System in the SAMP8 Mouse Model of Alzheimer’s Disease. Neural Plast..

[367] Liu P.-R., Cao F., Zhang Y., Peng S. (2019). Electroacupuncture Reduces Astrocyte Number and Oxidative Stress in Aged Rats with Surgery-Induced Cognitive Dysfunction. J. Int. Med. Res..

[368] Shi G.-X., Li Q.-Q., Yang B.-F., Liu Y., Guan L.-P., Wu M.-M., Wang L.-P., Liu C.-Z. (2015). Acupuncture for Vascular Dementia: A Pragmatic Randomized Clinical Trial. Sci. World J..

[369] Jia Y., Zhang X., Yu J., Han J., Yu T., Shi J., Zhao L., Nie K. (2017). Acupuncture for Patients with Mild to Moderate Alzheimer’s Disease: A Randomized Controlled Trial. BMC Complement. Altern. Med..

[370] Liang Z., Valla J., Sefidvash-Hockley S., Rogers J., Li R. (2002). Effects of estrogen treatment on glutamate uptake in cultured human astrocytes derived from cortex of Alzheimer's disease patients. J. Neurochem..

[371] Jiang X., Wu Q., Zhang C., Wang M. (2021). Homoharringtonine Inhibits Alzheimer’s Disease Progression by Reducing Neuroinflammation via STAT3 Signaling in APP/PS1 Mice. Neurodegener. Dis..

